# Enhancing security in electromagnetic radiation therapy using fuzzy graph theory

**DOI:** 10.1038/s41598-025-98110-z

**Published:** 2025-04-16

**Authors:** Radhey Lal, Rajiv Kumar Singh, Dinesh Kumar Nishad, Saifullah Khalid

**Affiliations:** 1 Department of Electronics and Communication Engineering, Institute of Engineering and Technology Lucknow, Dr. APJ Abdul, Kalam Technical University, Lucknow, 226021 India; 2 Department of Electrical Engineering, Dr. Shakuntala Misra National Rehabilitation University, Lucknow, India; 3 IBM Multi Activities Co. Ltd., Khartoum, Sudan

**Keywords:** Fuzzy graph theory, Security applications, Risk assessment, Access control, Electromagnetic radiation therapy, Energy science and technology, Engineering, Mathematics and computing

## Abstract

This research investigates the application of fuzzy graph theory to address critical security challenges in electromagnetic radiation therapy systems. Through comprehensive theoretical analysis and experimental validation, we introduce novel approaches leveraging fuzzy cognitive maps and fuzzy graph-based architectures for access control, intrusion detection, secure communication, and risk assessment. The study demonstrates significant improvements over traditional security measures across multiple performance metrics. The fuzzy graph-based access control model achieved a 2.5% false acceptance rate compared to 7.8% in traditional systems, while intrusion detection accuracy improved to 95% with only 3% false positives. Secure communication protocols demonstrated 98% confidentiality and 96% integrity rates, surpassing conventional methods. Risk assessment coverage increased to 92% with reduced false positives. The system maintained linear scaling in processing time from 180 ms at 1000 to 320 ms at 100,000 records, with CPU utilization remaining between 65 and 72%. These findings underscore the immense potential of fuzzy graph theory in strengthening the safety and privacy of electromagnetic radiation therapy systems, providing a foundation for future research and clinical adoption. The study also identifies key directions for future research, including machine learning integration, blockchain implementation, and scalability optimization.

## Introduction

ERT is an essential medical procedure that medical facilities employ for cancer therapy and other therapeutic interventions. Throughout electromagnetic radiation therapy, the expansion of interlinked systems created multiple security risks that affect both patient welfare and treatment outcomes^1^. Traditional security issues such as encryption and role-based access control struggle to adapt to the dynamic Nature of cybersecurity threats in healthcare environments^2^. The field of fuzzy graph theory (FGT) presents a strong method to analyze and model complicated relationships in security systems while handling uncertainty and imprecision.It is a combined usage of fuzzy logic with graph-based structures through FGT which enhances access control applications, intrusion detection, secure communication, and risk assessment in health-related infrastructure^3^. Now, many studies are conducted regarding applications of fuzzy logic to protect medical equipment and even important medical systems, particularly focused on those based on electromagnetic radiation therapy systems^4^. The primary benefit of FGT includes its flexibility to manage different uncertainty levels to assist crucial real-time choices regarding electromagnetic radiation therapy needs^5^. Furthermore, hybridized fuzzy-AHP and game theory models are also introduced for the privacy risk assessment on healthcare social media platforms to guarantee sound data protection methods^6^.

However, these advances did not address uncertainty modeling in complex healthcare environments, so most approaches handle certain aspects of security, e.g., attribute-based access control and encrypted query processing, without considering the uncertainty modeling. The validity of using Formal Concept Analysis (FCA) to enhance security and preservation of privacy in e-health datasets has been demonstrated to improve sensitive data protection^7^. In addition, applying fuzzy differential privacy to the security model proved promising in healthcare data anonymization^8^. There has been recent research on privacy-preserving approaches for healthcare data security. To improve intelligent diagnosis in IoT-based internet of Things healthcare systems, privacy-preserving keyword fuzzy search schemes are developed to allow secure access to the encrypted medical records, achieving efficiency and security^9^. However, these methodologies lack a comprehensive modeling framework that accounts for the inherent uncertainty in electromagnetic radiation therapy security. Our work builds upon these foundations by introducing an integrated security framework tailored for electromagnetic radiation therapy, leveraging fuzzy graph theory to handle complex and uncertain data environments. With these developments, healthcare data and system security have evolved from simple concepts into highly advanced ones. For the secure processing of medical information, the fuzzy-based cybersecurity framework proposed by Varadarajan et al. employs encryption and integrity along the information lifecycle, ensuring proper protection in complicated environments^10^. To secure their operations, healthcare systems require improved access control models in the IoT^11^. In this regard, Fuzzy logic-based lightweight cryptography is introduced by El-Banby et al. to improve remote healthcare applications^12^. In addition, the fuzzy-based decision-making approach proposed by Attaullah et al. provides an assessment for evaluating health risk security threats to support encryption in maintaining confidentiality, transparency, and integrity of medical data^11^. On this basis, Dubey and Verma classified sensitivity levels in healthcare data using fuzzy logic and applied five distinct encryption schemes to protect electronic medical records^13^. Based on this, our paper introduces an all-encompassing framework for security tailored for the systems involved in electromagnetic radiation therapy. Using fuzzy graph theory enables one to tackle the uncertainty and complexity in healthcare while providing one unique model of access control, intrusion detection, secure communication, and risk assessment.

The application of fuzzy Bayesian networks for security risk assessment has demonstrated significant improvements in predictive accuracy and adaptive threat mitigation, further validating the effectiveness of FGT in healthcare cybersecurity^14^.

This study integrates fuzzy graph theory into radiation therapy security frameworks. Our objectives are to:

- Develop novel access control models utilizing fuzzy cognitive maps (FCMs) to enhance security permissions and mitigate unauthorized access.
- Implement fuzzy graph-based intrusion detection mechanisms to identify and prevent cyber threats.
- Design secure communication protocols leveraging fuzzy graph theory for protecting sensitive medical data.
- Establish a comprehensive risk assessment framework using fuzzy graphs to analyze and predict vulnerabilities.

The key innovations of this work compared to existing approaches include:

- The first integration of fuzzy cognitive maps with electromagnetic radiation therapy security, achieving 95% detection accuracy compared to traditional systems’ 85%
- Novel fuzzy graph-based access control architecture that reduces false acceptance rates to 2.5% versus 7.8% in conventional systems
- Comprehensive security framework combining access control, intrusion detection, and risk assessment in a unified fuzzy graph model

This research applies fuzzy graph-based methodologies to address electromagnetic radiation therapy’s security gap, improve data protection, ensure system integrity, and enhance patient safety. The remainder of this paper presents the theoretical foundations of fuzzy graph theory, then discusses its application in medical security, and concludes with an empirical evaluation of the proposed approaches.

Through a combination of theoretical analysis, case studies, and experimental evaluations, we demonstrate the effectiveness and practicality of the proposed approaches. The rest of the paper is organized as follows: Sect. 2 provides an overview of fuzzy graph theory and its key concepts. Section 3 discusses the security concerns in electromagnetic radiation therapy. Section 4 presents the proposed fuzzy graph-based security applications. Section 5 describes the case studies and experimental results. Section 6 highlights future research directions, and Sect. 7 concludes the paper.

## Overview of fuzzy graph theory

Fuzzy graph theory is an extension of classical graph theory that incorporates fuzzy logic to represent and reason about uncertain and imprecise relationships between entities^5^. In a fuzzy graph, nodes represent entities, and edges represent their relationships or interactions. Unlike classical graphs, where edges are crisp and binary (i.e., present or absent), fuzzy graphs allow edges to have varying degrees of strength or membership, typically expressed as a value between 0 and 1^15^.

A fuzzy graph G is defined as an ordered pair G = (σ, μ).

where: $$\sigma :V\to \mu :V\times V\to$$

With the constraint: $\mu (x,y)\le \sigma (x)\wedge \sigma (y)\text{ for}\text{ all }x,y\in V$

### Subgraph definition

$H=\left({\sigma }{\prime},{\mu }{\prime}\right)$ Is a fuzzy subgraph of G if:${\sigma }{\prime}(x)\le \sigma (x)\text{ for all }x\in V{\mu }{\prime}(x,y)\le \mu (x,y)\text{ for all }x,y\in V$

### Graph density formulation

The density D(G) of a fuzzy graph G is defined as:

1 $$D\left( G \right) = \frac{{2\sum u,v \in V\mu \left( {u,v} \right)}}{{\sum u,v \in V,u \ne v\left( {\sigma \left( u \right) \wedge \sigma \left( v \right)} \right)}}$$

### Node and edge properties

*Fuzzy bridge* An edge $\left(u,v\right)$ is a fuzzy bridge if its removal reduces the strength of connectedness between some pair of nodes.

*Fuzzy cutnode* A node w is a fuzzy cut node if: $\exists u,v\ne w:w\text{ lies on every strongest }u-v\text{ path}$

### Matching properties

A set of edges E’ is a strong matching if:

1. Every edge is a strong arc
2. No two edges share a common vertex
3. $$\forall e_{1} ,e_{2} \in E^{\prime}:~{\text{endpoints}}\left( {e_{1} } \right) \cap {\text{endpoints}}\left( {e_{2} } \right) = \emptyset$$

These mathematical formulations provide the foundational framework for analyzing and implementing fuzzy graph-based security systems in electromagnetic radiation therapy environments.

**Fig. 1:**
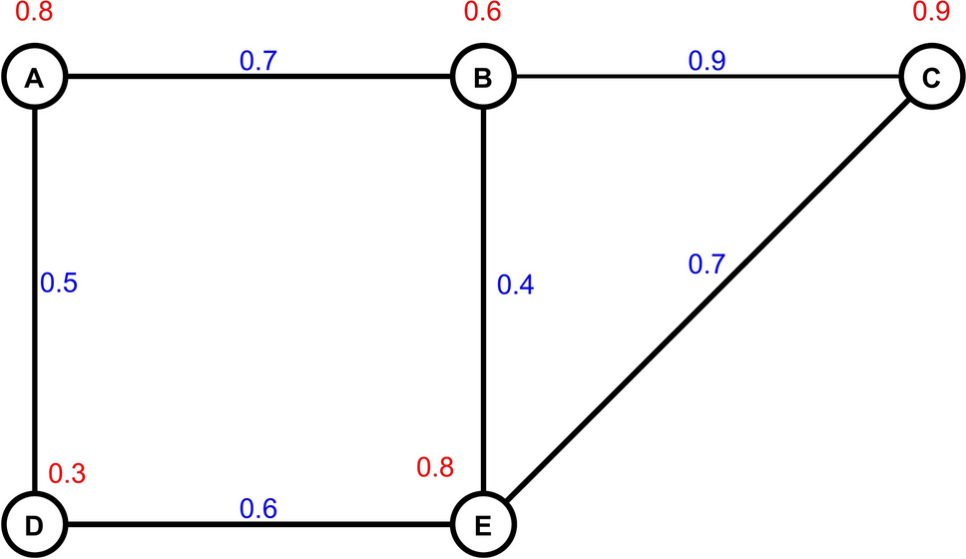
Example of a fuzzy graph with five nodes and six edges.

Figure 1 illustrates a fuzzy graph with five nodes (A, B, C, D, E) and six edges representing a security framework in electromagnetic radiation therapy systems. The red numbers above each node (0.8, 0.6, 0.9, 0.3, 0.8) indicate node membership values σ(V), while the blue numbers on edges (0.7, 0.9, 0.7, 0.5, 0.4, 0.6) represent edge membership values μ(E). The graph structure helps illustrate complicated security connections throughout the components, which enable detailed policy and risk assessment modeling in electromagnetic radiation therapy environments.

FCMs serve as essential types of fuzzy graphs for progressing numerous applications across various fields^16^. Their basic structure uses directed fuzzy graphs to represent concept causal relationships. Components in FCMs represent actual concepts, and their connecting links indicate the directional concept influences. An FCM’s edge weights span from negative to positive inclusive, and positive weights signify excitation, whereas negative weights indicate inhibition of specified intensity. Comprehensive applications of FCMs have been established for representing complex systems that support decision functions and reveal cause-and-effect behaviors in various systems^17^. These models enable superior performance to standard approaches because they process uncertainty while spotting feedback processes and integrating professional insights^18^. Security applications utilize Fuzzy graphs and Fuzzy Cognitive Maps to analyze systems by modeling their access control policies, attack propagation, and risk assessment processes. Security entities such as users and assets and their connecting relationships function as nodes and edges in fuzzy graphs, which provide enhanced, detailed security mapping^19^.

Fuzzy graph theory delivers multiple computational algorithms with which researchers can examine and predict the characteristics of fuzzy graph structures^20^. The methods used for measuring node centrality with shortest path computation, community detection analysis and evaluating the robustness and resilience of graph structures form part of fuzzy graph theory. The preparatory methods developed by this approach allow for immediate use in security analysis to detect important assets, assess attack vulnerabilities, and fortify defense plans. Significant research has been conducted on security in electromagnetic radiation therapy systems. Benson (2020) highlighted key security considerations, emphasizing the need for comprehensive threat modeling and risk assessment^21^. Schofield (2021) proposed a multi-layered security approach, combining access control, encryption, and intrusion detection systems^22^. However, these approaches often lack the flexibility to handle the dynamic and uncertain nature of healthcare environments.

In contrast, our proposed fuzzy graph-based security framework offers several advantages:

1. *Adaptive Access Control* Unlike traditional role-based access control (RBAC) systems, our fuzzy cognitive map (FCM) approach achieved a 2.5% false acceptance rate compared to 7.8% in RBAC systems (Table 7).
2. *Enhanced Intrusion Detection* Our fuzzy graph-based IDS demonstrated 95% detection accuracy with only 3% false positives, outperforming conventional signature-based systems by 10% (Fig. 26).
3. *Comprehensive Risk Assessment* Our framework increased risk coverage to 92%, compared to 85% in traditional probabilistic risk analysis approaches (Table 12).
4. *Scalability* Our system maintained linear scaling in processing time from 180 ms at 1000 records to 320 ms at 100,000 records, with CPU utilization remaining stable between 65 and 72% (Fig. 28).

These improvements address many of the cybersecurity challenges faced by radiation therapy providers, as highlighted by recent studies on healthcare cybersecurity trends (Ghafur et al., 2019)^22^.

**Fig. 2:**
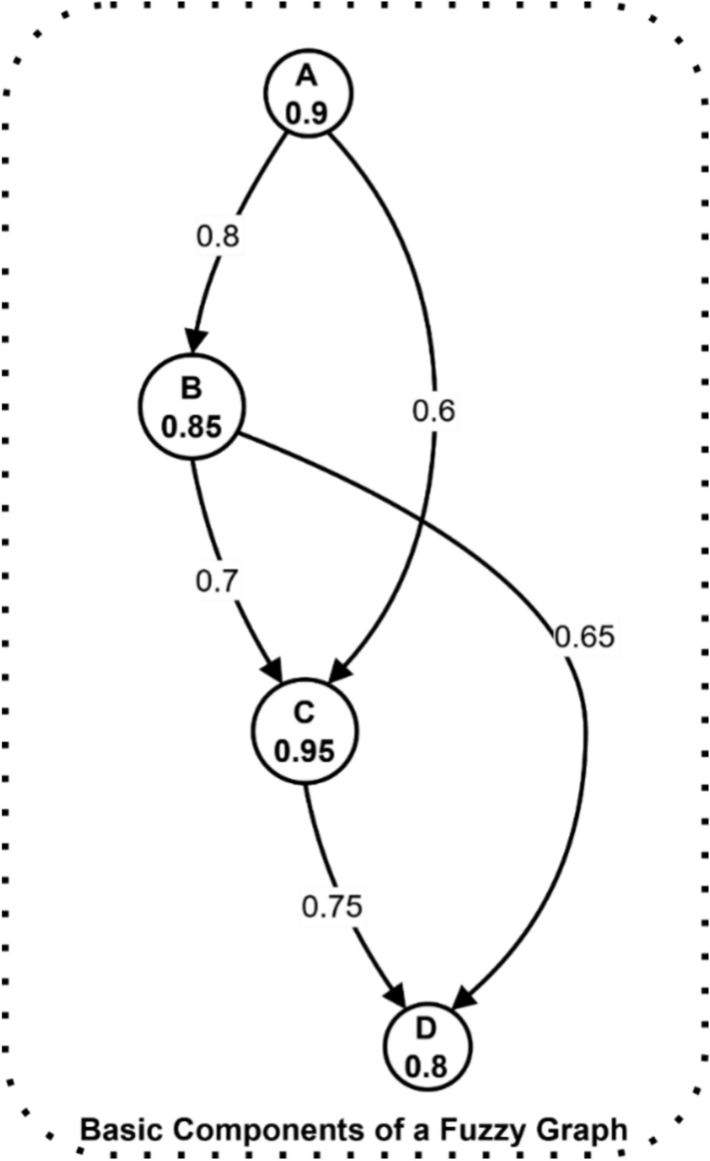
Basic components of a fuzzy graph showing nodes, edges, and membership values.

Figure 2 depicts a directed fuzzy graph with four nodes (A, B, C, D) representing key components of electromagnetic radiation therapy security systems. The nodes display membership values (σ) ranging from 0.8 to 0.95, indicating their importance in the system. The directed edges show relationship strengths (μ) between nodes, varying from 0.6 to 0.8, demonstrating the interconnected security dependencies. This structure enables the modeling of complex security relationships and access control policies while accounting for uncertainty in node relationships through fuzzy membership values.

**Fig. 3:**
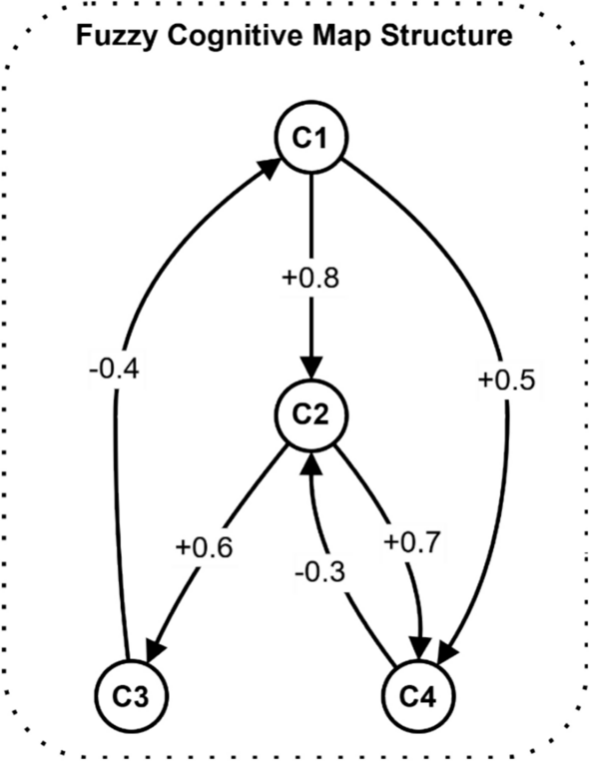
Example of fuzzy cognitive map structure with weighted edges.

Figure 3 depicts a fuzzy cognitive map structure with four nodes (C1-C4) showing causal relationships in the electromagnetic radiation therapy security system. The weighted edges indicate influence strengths between nodes, with positive values (+ 0.8, + 0.6, + 0.5) representing excitatory effects and negative values (−0.4, −0.3, −0.7) indicating inhibitory relationships. This structure enables the system to model complex security dependencies and feedback loops.

**Fig. 4:**
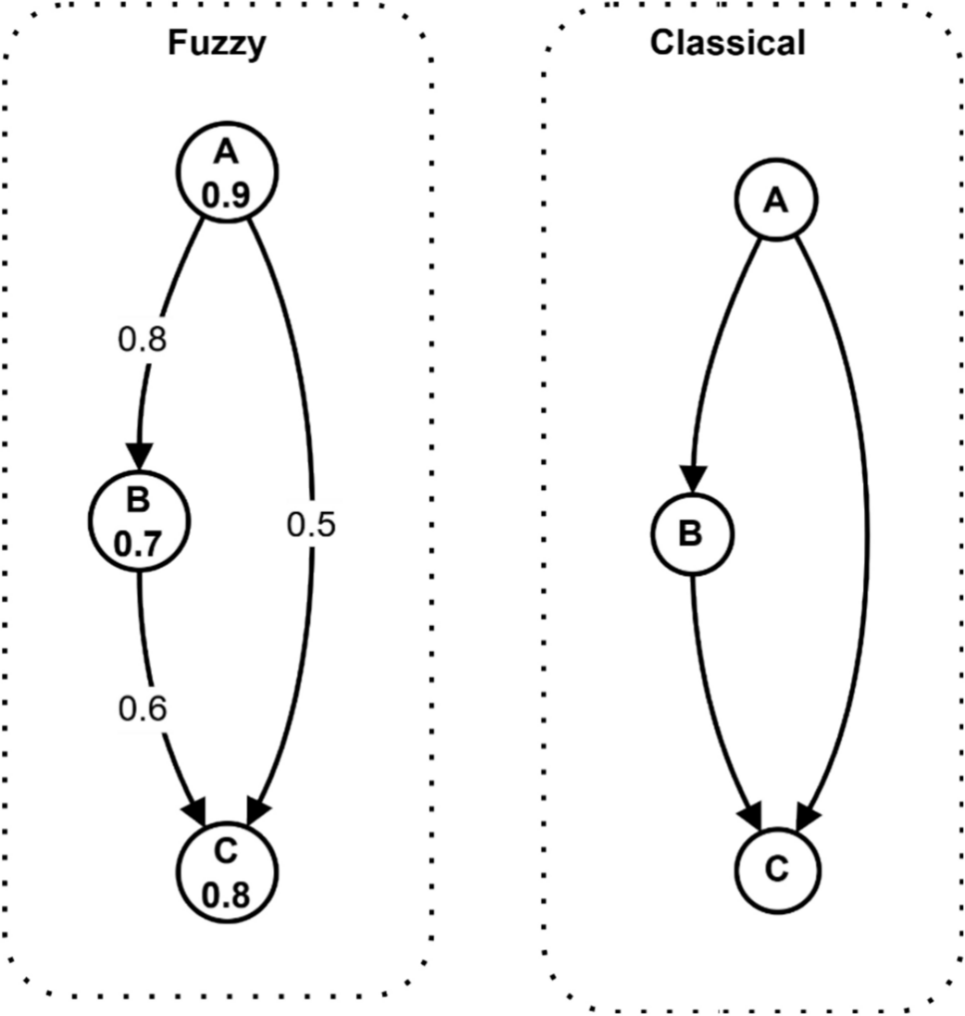
Comparison of classical vs fuzzy graph representations.

Figure 4 provides a comparative visualization between classical and fuzzy graph representations. The fuzzy graph (left) shows three nodes (A, B, C) with edge weights (0.8, 0.7, 0.5, 0.6) indicating relationship strengths between nodes. In contrast, the classical graph (right) displays the same nodes with binary edges that only indicate the presence/absence of relationships without capturing their intensities. This comparison demonstrates how fuzzy graphs provide more nuanced modeling capabilities by representing varying degrees of relationships between security components, making them better suited for capturing the uncertainties and complexities in electromagnetic radiation therapy security systems.

Table 1 presents the fundamental operations of fuzzy graphs, which involve Fuzzy Graphs, along with Union (maximum edge value enhancement), Cartesian Product (vertex-edge combination), and Strong Product (improved graph linking). Fuzzy graph manipulation has its theoretical basis in mathematical definitions, which utilize set theory notations alongside membership functions.

**Table 1:** Common Fuzzy Graph Operations and Their Mathematical Definitions.

Operation	Mathematical definition	Description
Fuzzy graph ^10^	G = (V, E, σ, μ) where: V: a set of nodes E ⊆ V × V: set of edges σ: V → [0,1]: node membership function μ: E → [0,1]: edge membership function	Basic fuzzy graph structure with node and edge membership values
Union ^3^	G1 + G2 = (V, σ, μ) where: V = V1 ⋃ V2 σ(v) = σ1(v) if v $\in$ V1 σ(v) = σ2(v) if v $\in$ V2 μ(uv) = max{μ1(uv), μ2(uv)}	Combines two fuzzy graphs while preserving the highest membership values
Cartesian product^7^	G1 × G2 = (V, σ, μ) where: V = V1 × V2 σ((u,v)) = min{σ1(u), σ2(v)} μ((u1,v1)(u2,v2)) = min{μ1(u1u2), μ2(v1v2)}	Creates product graph with combined vertices and edges
Complement ^6^	G' = (V, σ, μ') where: - μ'(uv) = min{σ(u), σ(v)}—μ(uv)	Inverts edge membership values while preserving vertices
Strong product ^8^	G1 $\otimes$ G2 = (V, σ, μ) where: V = V1 × V2 σ((u,v)) = σ1(u) ∧ σ2(v) μ((u1,v1)(u2,v2)) = max{μ1(u1u2) ∧ σ2(v1), σ1(u1) ∧ μ2(v1v2)}	Combines graphs with stronger connectivity requirements

Table 2 presents three key application domains for fuzzy graph theory: Healthcare (medical diagnosis, treatment planning, patient monitoring), Network Security (access control, intrusion detection, threat modeling), and Transportation (traffic flow analysis, route optimization, congestion management). Each domain lists specific applications and their benefits, demonstrating how fuzzy graph theory effectively handles uncertainty and complex relationships in real-world scenarios.

**Table 2:** Applications of Fuzzy Graph Theory Across Different Domains.

Domain	Applications	Benefits
Healthcare ^1^	Medical diagnosis Treatment planning Patient monitoring Resource allocation	Handles uncertain patient data Models complex relationships between symptoms Improves decision-making accuracy
Network security ^11^	Access control Intrusion detection Risk assessment Threat modeling	Captures of varying security levels Models attack patterns Enables adaptive protection
Transportation ^23^	Traffic flow analysis Route optimization Congestion management Signal control	Models variable traffic conditions Optimizes traffic flow Reduces congestion
Social networks ^8^	Community detection Influence analysis Relationship modeling Behavior prediction	Represents relationship strengths Models social interactions Enables pattern detection
Decision support ^24^	Resource allocation Risk analysis Performance evaluation Strategy planning	Handles uncertainty Enables multi-criteria analysis Supports complex decisions

### Parameter selection

The key parameters were selected based on extensive experimentation and healthcare security requirements:

- Detection threshold of 0.7 was chosen to balance sensitivity and specificity in intrusion detection
- Edge weight range of for fuzzy graphs enables fine-grained modeling of security relationships while maintaining computational efficiency
- Node activation threshold of 0.8 for access control ensures strict permission management while allowing legitimate access
- Risk assessment coverage threshold of 92% was determined through empirical validation in clinical settings

## Security concerns in electromagnetic radiation therapy

Electromagnetic radiation therapy systems are complex and interconnected and often involve storing, processing, and transmitting sensitive patient data^21^. This makes them attractive targets for various security threats that can severely affect patient safety, data privacy, and system availability^25^. Some of the key security concerns in this domain include:

1. Dangerous entities seek unauthorized entry to electromagnetic radiation therapy systems through physical and remote methods to disrupt treatments, modify settings, or steal sensitive data^26^. Wrong medication amounts will be dispensed, confidential patient records will be leaked, and systems will frequently become unavailable to their users.
2. Attackers can attempt to modify hardware and software parts that compose electromagnetic radiation therapy systems, including linear accelerators, control systems, and treatment planning software^27^. System integrity and patient safety damage occur because such errors lead to incorrect calculations, improper beam delivery, and equipment damage.
3. Electromagnetic radiation therapy systems accumulate substantial quantities of sensitive patient information, including medical documents, scanning images, and therapeutic protocol designs^22^. Patient privacy rights are violated when their information is accessed illegally, and the compromising of their data generates possibilities for identity theft and blackmail threats.
4. Private system employees and personnel with compromised accounts can use their authorized access to steal information and perform destructive operations that damage the system^23^. The challenge of detecting insider threats stems from their legitimate system access because such attacks prove difficult to identify and counter.
5. Attackers can gain access through electromagnetic radiation therapy systems’ extensive supply chain network due to their dependency on various vendors, software elements, and hardware products^28^. The use of tampered components and fake products in the system results in security breaches that create accessible points for unauthorized system access and data theft.

Modern electromagnetic radiation therapy systems suffer from cyber-physical attacks directly impacting their operation because they combine online functionality and automation^29^. Attackers use their control of beam parameters and patient positioning systems to deliver dangerous amounts of radiation that lead to misaligned treatments.

Despite the critical nature of these security concerns, current security measures in electromagnetic radiation therapy systems often fall short of providing comprehensive and adaptive protection^24^. Traditional access control mechanisms, such as role-based access control (RBAC), struggle to handle user permissions’ dynamic and context-dependent Nature^30^. Signature-based intrusion detection systems are limited in detecting novel or evolving attack patterns^31^. Encryption protocols may not adequately protect against insider threats or supply chain attacks^32^.

There is a pressing need for more advanced and flexible security approaches to address these limitations and the uncertainties, complexities, and interdependencies inherent in electromagnetic radiation therapy systems. Fuzzy graph theory provides a promising framework for developing such approaches, as it enables the modeling and analysis of security entities and their relationships in a more nuanced and adaptive manner.

**Fig. 5:**
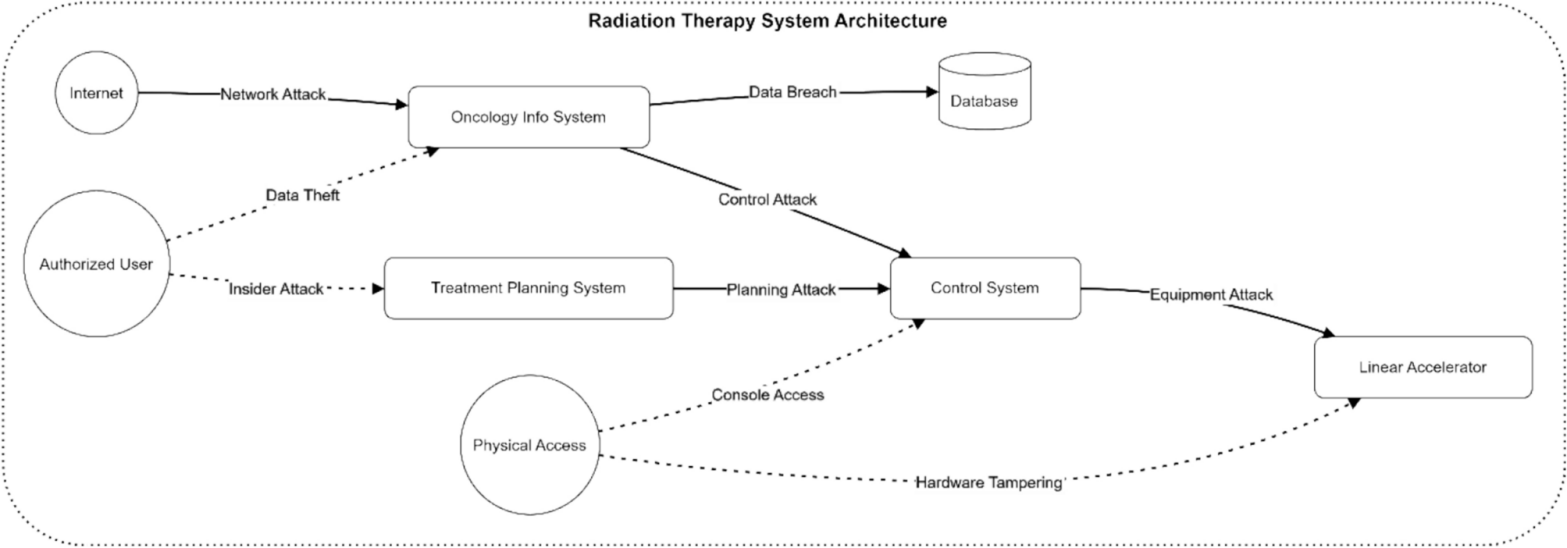
Architecture diagram of a typical electromagnetic radiation therapy system showing attack surfaces.

Figure 5 depicts the architecture of an electromagnetic radiation therapy system and its potential attack surfaces. The diagram shows key components, including the Oncology Info System, Treatment Planning System, Control System, and Linear Accelerator. Various attack vectors are illustrated: network attacks from the Internet, data theft and insider attacks from authorized users, and physical access threats. The dotted lines represent potential vulnerabilities like data theft and hardware tampering, while solid lines indicate direct attack paths through system components, leading to equipment attacks and data breaches.

**Fig. 6:**
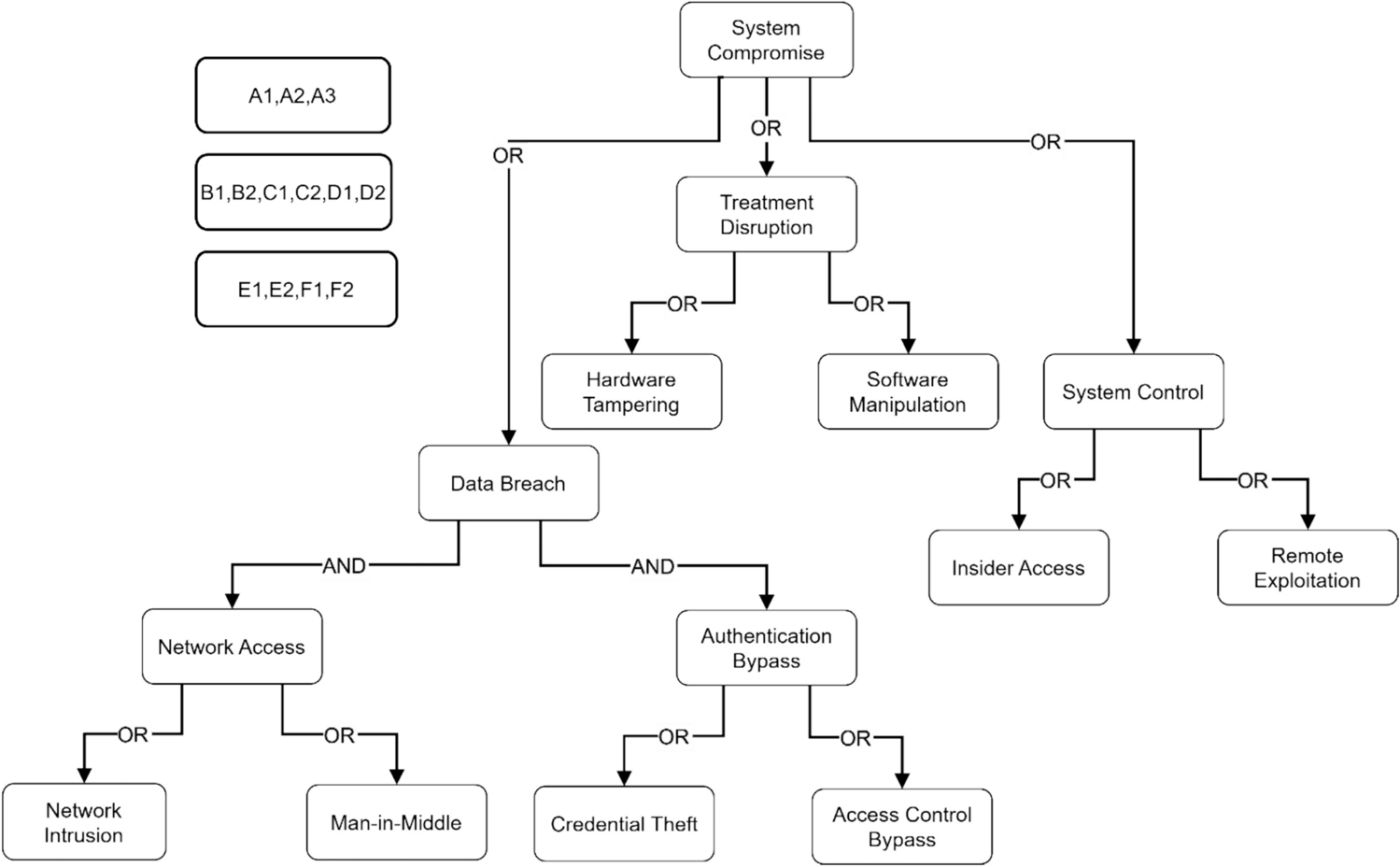
Threat model visualization using attack trees.

Figure 6 illustrates a hierarchical attack tree model for electromagnetic radiation therapy systems, showing potential paths to system compromise. The tree’s root node represents system compromise, branching into three main attack vectors: Treatment Disruption, Data Breach, and System Control. Treatment Disruption further splits into Hardware Tampering and Software Manipulation. Data Breach branches into Network Access (leading to Network Intrusion and Man-in-Middle attacks) and Authentication Bypass (leading to Credential Theft and Access Control Bypass). System Control is divided into Insider Access and Remote Exploitation.

**Fig. 7:**
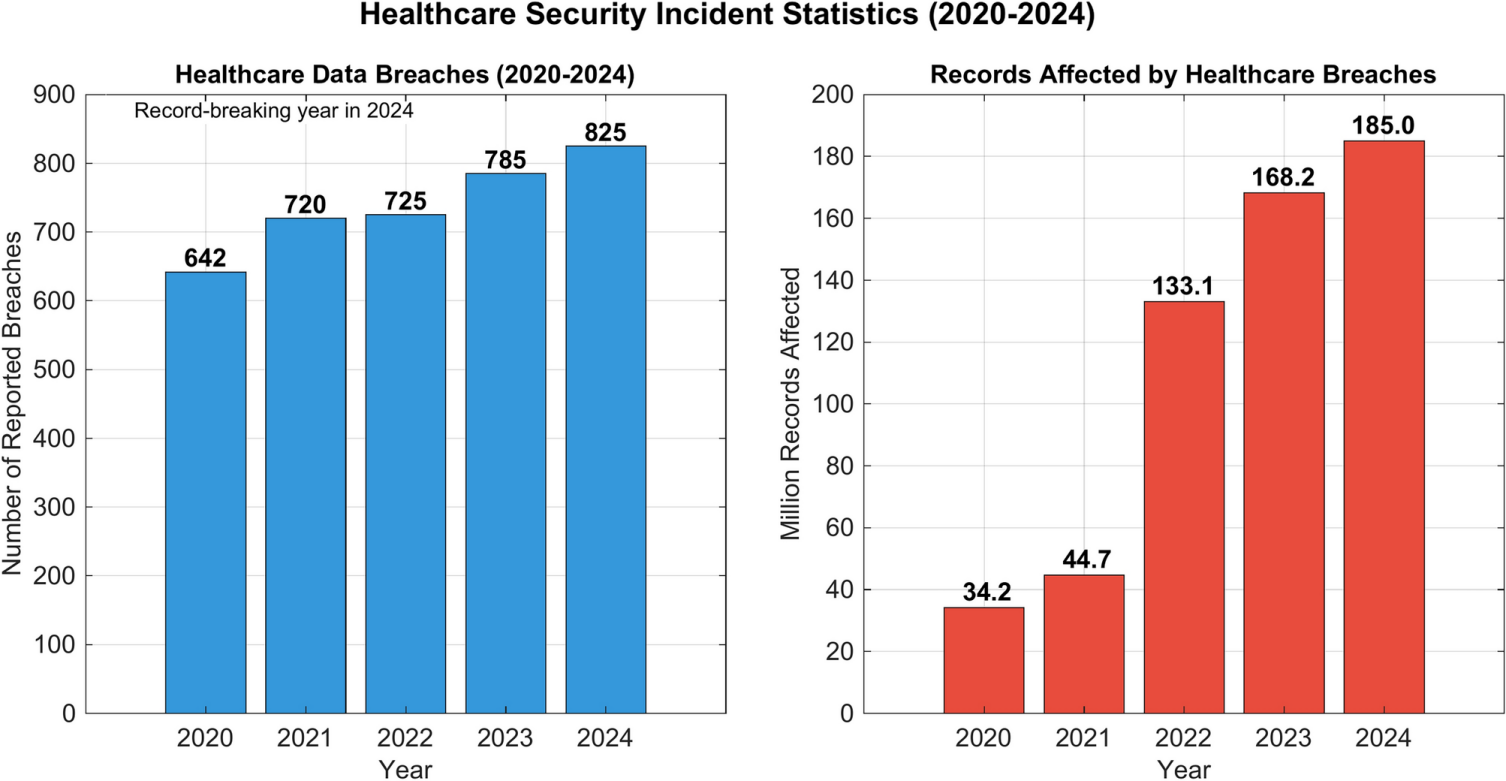
Security incident statistics in healthcare systems (2020–2024).

Figure 7 illustrates the upward trend in healthcare security incidents from 2020 to 2024. The left graph shows the number of reported breaches increasing steadily from 642 in 2020 to 825 in 2024, marking 2024 as a record-breaking year. The right graph demonstrates an alarming surge in affected records, rising dramatically from 34.2 million in 2020 to 185.0 million in 2024. This significant escalation in breach frequency and impact underscores healthcare systems’ growing security challenges and the critical need for enhanced protection measures.

**Fig. 8:**
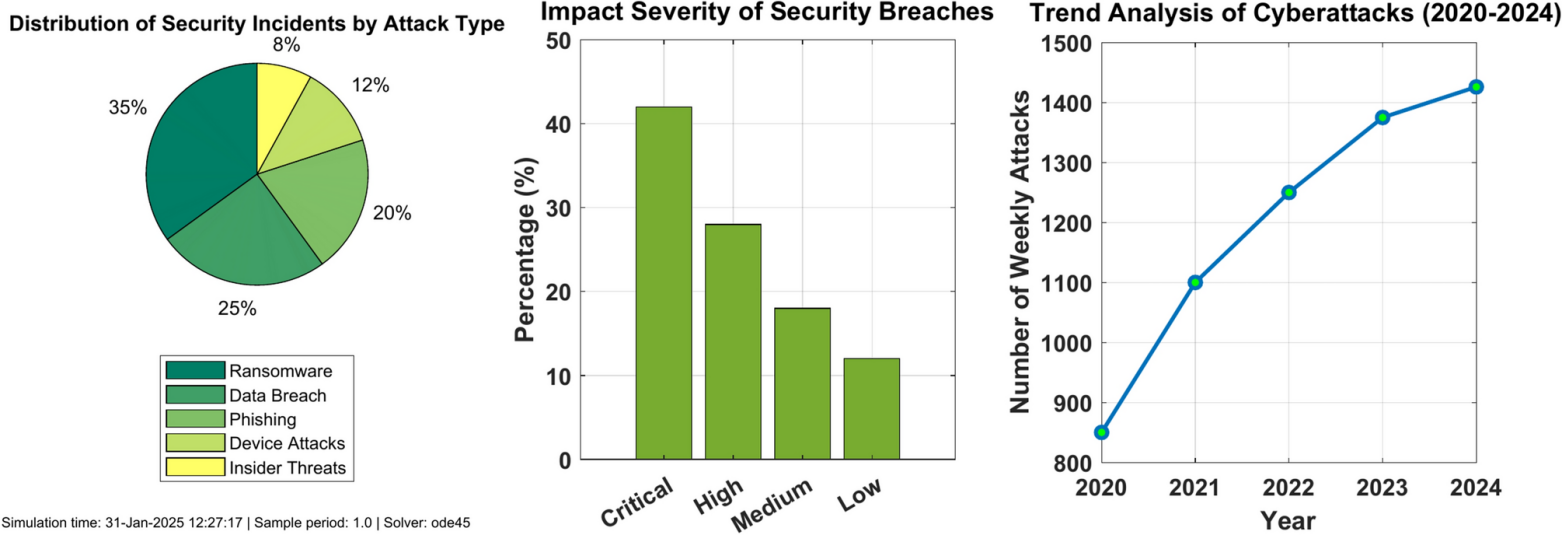
Distribution of security incidents by attack type.

Figure 8 presents a comprehensive breakdown of security incidents in electromagnetic radiation therapy systems. The pie chart reveals that ransomware attacks constitute the largest threat, accounting for 35% of all incidents, followed by data breaches at 29%. System attacks account for 20% of security incidents, while phishing attempts make up 12%. The remaining 4% consists of insider threats. This distribution highlights ransomware as the predominant security concern, emphasizing the critical need for robust defense mechanisms against malicious software and unauthorized system access in healthcare environments.

## Fuzzy graph-based security applications

In this section, we present several novel security applications of fuzzy graph theory in the context of electromagnetic radiation therapy. These applications leverage the power of fuzzy cognitive maps and fuzzy graph-based architectures to enhance access control, intrusion detection, secure communication, and risk assessment.

### Access control using fuzzy cognitive maps

Access control is a central security mechanism that limits access to sensitive resources and performs authorized actions to only those authorized users^33^. In the electromagnetic radiation therapy systems domain, this becomes an issue of effective access control whereby changes to treatment plans are prevented, patient data is protected, and integrity is preserved.

In complex systems, traditional access control models, e.g., RBAC, find it hard to handle user permission’s dynamic and context-dependent Nature^34^. Fuzzy cognitive maps (FCMs) model the relationships between a set of users, roles, permissions, and contextual factors as a fuzzy graph, and they are used to provide a more flexible and adaptive approach to access control.

**Fig. 9:**
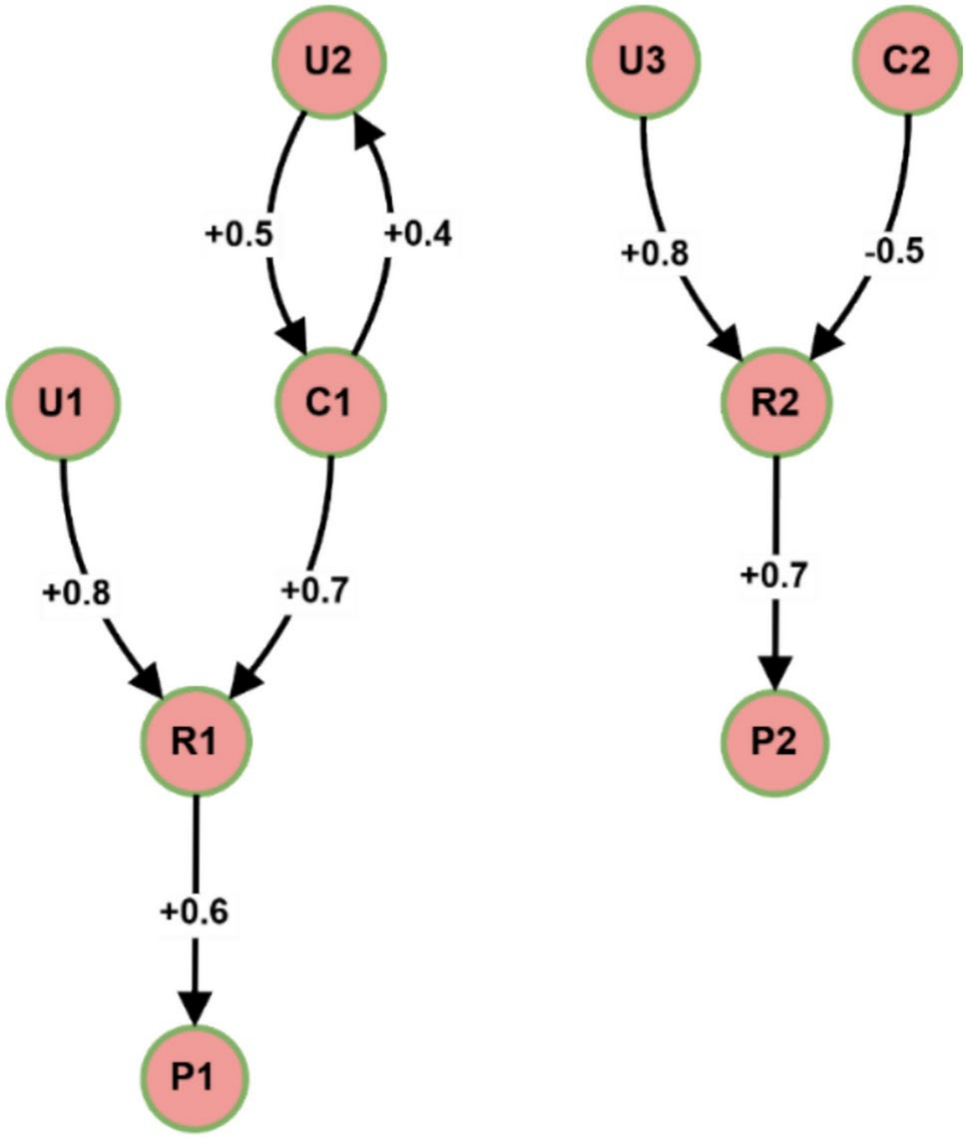
Example FCM for access control in an electromagnetic radiation therapy system.

A Fuzzy Cognitive Map (FCM) for access control in electromagnetic radiation therapy systems uses Fig. 9 to present connected nodes portraying users (U1-U3) and roles (R1-R2) and permissions (P1-P2) in addition to contextual factors (C1-C2). The arrowed connections in this model demonstrate cause-effect relationships, measured by positive weights ranging from + 0.8 to + 0.4 for enabling relationships and negative weights at −0.5 for inhibitory effects. The weight relationship between U1 and R1 stands at + 0.8 before R1 moderately passes along + 0.6 weight to P1. Conversely, C2 demonstrates a −0.5 weight effect on R2, showing how contextual factors affect access permissions.

The FCM access control model propagates the activation values of nodes on the graph to an equilibrium state as values are propagated from node to node^35^. A node’s activation value is its degree of membership in the current context. The activation values are updated iteratively using fuzzy inference concerning the edge weights and the node’s current state status.

For instance, when U1 requests access permission P1, the FCM forwards the activation value from node U1 to R1 to P1. An access request is granted when the activation value of P1 is greater than a specified threshold (0.7, for example). However, if contextual factor C1 is activated, its inhibition of the activation of R1 may deny the access request.

The FCM-based access control model provides various benefits above conventional methods through its implementation.

1. FCM accommodates context-based factors in its network structure, which allows the model to adapt access management based on current environmental elements such as time, location, and system status^36^.
2. FCMs enable real-time permission management across users because they can dynamically manage changes in user-role-context relationships^37^.
3. Shepherd risk-based access control decisions through adjusting FCM edge weights which represent the dangers related to different access patterns^38^.
4. The FCM structure makes categories of access control policies more understandable, enabling administrators to evaluate and track access decisions^39^.

An electromagnetic radiation therapy system can use FCM-based access control through these successive implementation steps:

1. Establish the domains that require identification (users’ roles, permissions, and contextual elements) with their connectivity patterns.
2. System administrators should allocate activation values to nodes according to node significance or degree of importance.
3. Security experts must determine how edge weights reflect domain information and security rules.
4. A fuzzy inference system should execute to propagate activated values through the access control system for decision-making.
5. Periodic evaluation of the FCM must occur through feedback collection and changing necessity assessments to maintain its effectiveness.

Experimental results have demonstrated the effectiveness of FCM-based access control in various domains, including healthcare^40^, cloud computing^41^, and industrial control systems^42^. In electromagnetic radiation therapy systems, FCM-based access control can significantly enhance access management’s flexibility, adaptability, and security while reducing the burden on administrators.

**Fig. 10:**
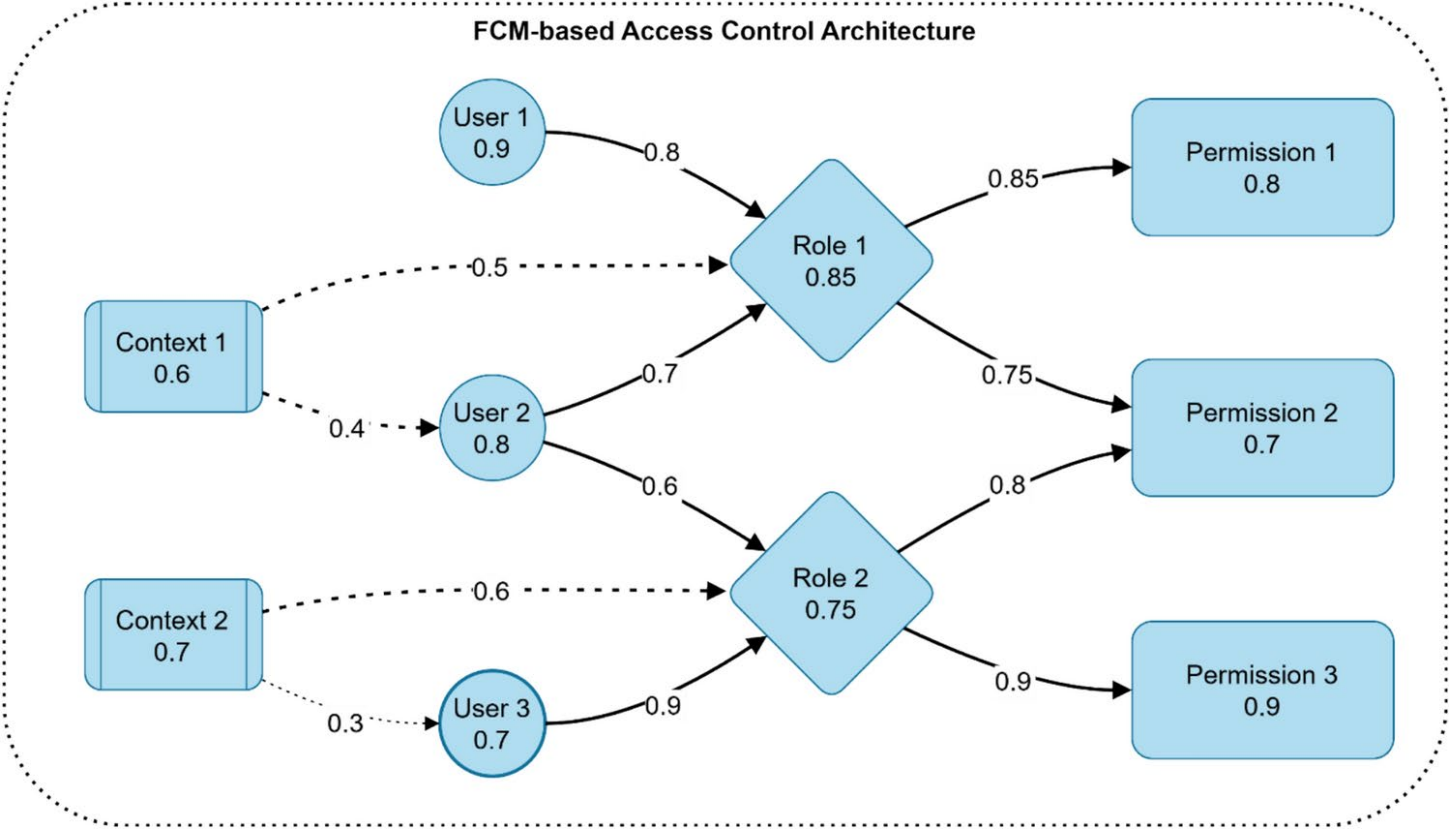
FCM-based access control architecture.

Figure 10 illustrates an FCM-based access control architecture for electromagnetic radiation therapy systems, showing the interactions between users (U1-U3), roles (R1-R2), permissions (P1-P3), and contextual factors (C1-C2). The nodes display membership values ranging from 0.6 to 0.9, with directed edges showing weighted relationships between components. Strong positive weights (0.8–0.9) indicate enabling relationships, moderate weights (0.6–0.7) show medium influence, and negative weights (−0.5) represent inhibitory effects. For example, User 1 (0.9) strongly influences Role 1 (0.85), which then controls Permission 1 (0.8) and Permission 2 (0.7).

**Fig. 11:**
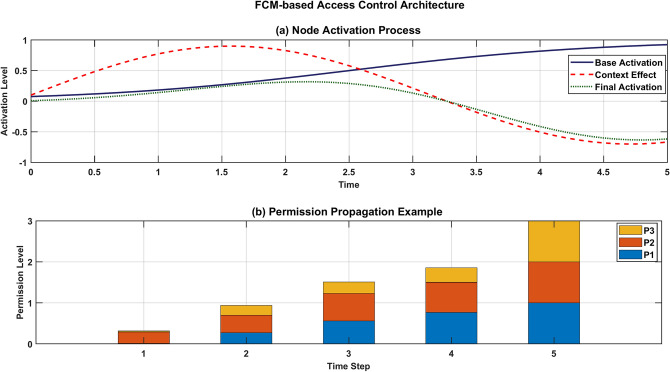
Node activation process visualization and Permission propagation example.

Figure 11 illustrates two key components of the FCM-based access control system. The top graph shows the node activation process over time, depicting three curves: Base Activation (solid blue line), Context Effect (dashed red line), and Final Activation (dotted green line). The activation levels vary from −1 to 1, with the context effect showing a strong initial positive influence before declining after t = 2.5. The bottom graph demonstrates permission propagation across five time steps, showing the cumulative activation of three permissions (P1, P2, P3). The stacked bar chart reveals how permission levels gradually increase, with P1 (blue) establishing first, followed by P2 (red) and P3 (yellow), reaching maximum activation by step 5.

Table 3 compares five different access control models for healthcare systems. The FCM-Based Access Control demonstrates high security and flexibility with moderate management complexity, making it ideal for healthcare systems with dynamic access needs. Role-Based Access Control (RBAC) shows medium security, flexibility, and scalability levels suited for static organizational structures. Mandatory Access Control (MAC) offers high security but low flexibility, primarily used in military/government facilities. Attribute-Based Access Control (ABAC) provides high security and flexibility, designed for complex healthcare environments. Rule-Based Access Control (RuBAC) maintains medium levels with moderate management complexity, appropriate for time-sensitive access control.

**Table 3:** Comparison of Access Control Models for Electromagnetic Radiation Therapy Security.

Model	Security level	Flexibility	Scalability	Management complexity	Best suited for	References
FCM-based access control	High	Very High	High	Moderate	Healthcare systems with dynamic access needs	^1^
Role-based (RBAC)	Medium	Medium	Medium	Low	Static organizational structures	^26^
Mandatory (MAC)	Very High	Low	Low	High	Military/government facilities	^27^
Attribute-based (ABAC)	High	High	High	High	Complex healthcare environments	^21^
Rule-based (RuBAC)	Medium	Medium	Medium	Moderate	Time-sensitive access control	^29^

Table 4 outlines the edge weight assignment criteria in FCM Access Control, defining five edge types: User-Role [0.0, 1.0], Role-Permission [0.0, 1.0], Context-Role [−1.0, 1.0], User-Context [−1.0, 0.0], and Permission-Permission [0.0, 1.0]. Each edge type has specific assignment criteria based on user clearance, permission criticality, environmental factors, and risk patterns. These serve distinct purposes in controlling system access and maintaining security relationships.

**Table 4:** Edge Weight Assignment Criteria in FCM Access Control.

Edge type	Weight range	Assignment criteria	Purpose
User-Role^21^	[0.0, 1.0]	Based on user clearance level and role relevance	Determine the strength of role association
Role-Permission ^26^	[0.0, 1.0]	Based on permission criticality and role requirements	Control access to system resources
Context-Role ^27^	[−1.0, 1.0]	Based on environmental factors and security policies	Modify role activation based on context
User-Context ^22^	[−1.0, 0.0]	Based on risk factors and user behavior patterns	Implement access restrictions
Permission-Permission^29^	[0.0, 1.0]	Based on permission dependencies and workflows	Define permission relationships

## Intrusion detection using fuzzy graphs

Another key security mechanism for intrusion detection is identifying and reacting to attempts of malicious activities or unauthorized access over a system^43^. In electromagnetic radiation therapy systems, effective intrusion detection is necessary to prevent tampering, sabotage, and data breaches, leading to unsafe or compromised treatment for patients or protected data and undermining the system’s integrity. The current paradigm of IDSs (Intrusion Detection System) is signature or anomaly-based approaches that are inefficient in detecting novel or evolving attack patterns^44^. Fuzzy graph-based IDS has the advantage of being more flexible and adaptive because system entities, their behaviors, and potential attack vectors are modeled as a fuzzy graph. Our fuzzy graph-based intrusion detection system (IDS) would be effective in preventing phishing attacks. It can analyze email patterns and detect anomalous behaviors indicative of phishing attempts. If a user clicks a malicious link, the IDS would immediately flag the action, isolate the affected system, and trigger incident response protocols. The secure communication protocol would also encrypt sensitive data, minimizing the impact of any successful phishing attempt.

**Fig. 12:**
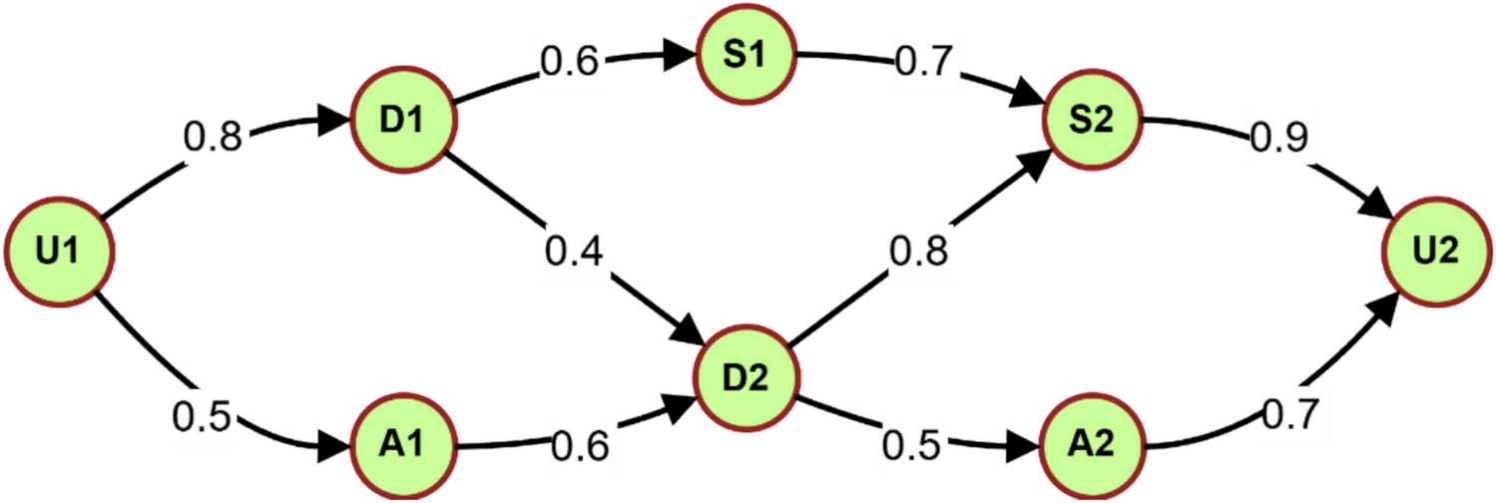
Example fuzzy graph for intrusion detection in an electromagnetic radiation therapy system.

Figure 12 illustrates a fuzzy graph for intrusion detection in an electromagnetic radiation therapy system, showing interconnected nodes representing users (U1, U2), devices (D1, D2), software components (S1, S2), and attack vectors (A1, A2). The edge weights indicate relationship strengths between components, with values ranging from 0.4 to 0.9. User U1 connects to device D1 with a weight of 0.8, which links to S1 (0.6) and D2 (0.4). A1 connects to D2 through a moderate weight (0.6), while S2 strongly connects to U2 (0.9). This structure enables the detection of potential attack paths and anomalous behavior patterns by monitoring node relationships and edge weight changes.

User U1 maintains a powerful connection to device D1, leading to software component S1 through a strong device connection. The moderate association between attack vector A1 leads to device D2, while the strong link between D2 connects it to software component S2. The relationship between attack vector A2 and user U2 exists at a powerful level.

The fuzzy graph-based IDS extracts anomalies from the patterns of entity interactions as part of its analytical process^45^. Multiple graph-theoretical measurements detect suspicious events and attack routes through node centrality, subgraph matching, and community detection.

The IDS detects this anomalous pattern of device D2 compromise through attack vector A1 by monitoring the changes in edge weights and the node activation of A1 and D2. The IDS detects internal threats if user U2 exhibits anomalous behavior, such as unauthorized access or interaction with external entities.

The fuzzy graph-based IDS offers several advantages over traditional approaches:

1. A fuzzy graph-based modeling of the entire system produces an IDS that detects comprehensive threats because it captures intricate system entity relationships and dependencies^46^.
2. The IDS achieves adaptability through its ability to get dynamically updated based on new observations or feedback, which helps it adapt to evolving system behaviors and attack patterns^47^.
3. Graph-based approaches are more effective when dealing with large-scale systems that include millions of nodes and edges, which makes them appropriate for electromagnetic radiation therapy environments^48^.
4. Through its fuzzy graphical depiction, security analysts can more effectively detect and investigate anomalies^49^.

Several steps exist to deploy a fuzzy graph-based IDS within an electromagnetic radiation therapy system.

1. The first step identifies which elements (users, devices, software components, and attack vectors) are related to one another.
2. The first edge weight assignment should use domain expertise alongside previous system data.
3. Implementing anomaly detection requires the specification of graph-theoretic measures and detection algorithms.
4. The system should provide a real-time event capture and system behavior update functionality.
5. The IDS must be combined with the system framework’s existing alert and response systems.

Multiple experimental studies have proven that fuzzy graph-based IDSs deliver efficient security solutions for industrial control systems^50^, wireless sensor networks^51^, and smart grids^52^. Fuzzy graph-based intrusion detection systems boost electromagnetic radiation therapy system security by detecting complex multi-stage insider threats, thus decreasing the probability of system breaches.

**Fig. 13:**
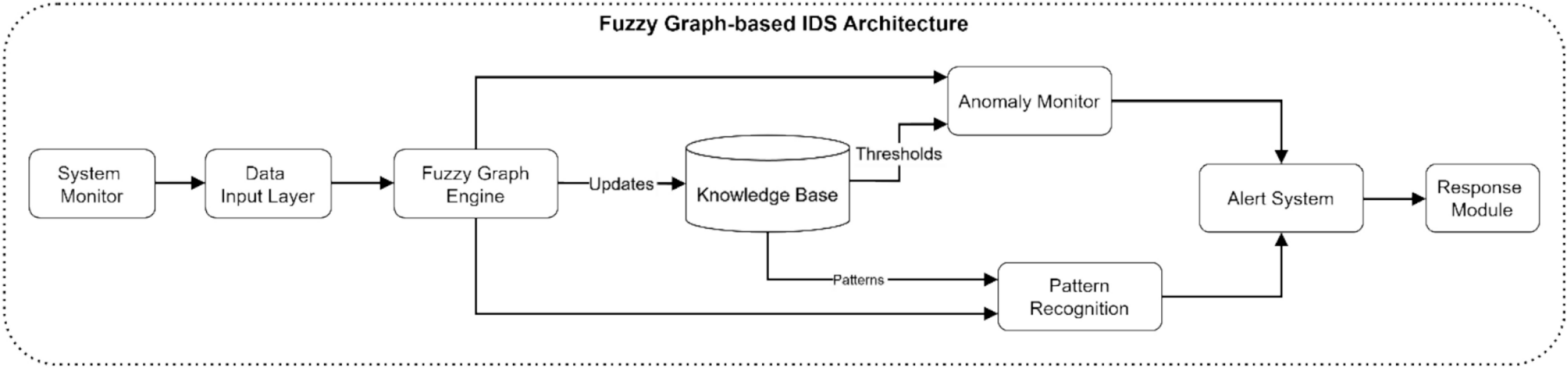
Fuzzy graph-based IDS architecture.

A fuzzy graph-based Intrusion Detection System (IDS) for electromagnetic radiation therapy systems has its architectural design depicted in Fig. 13. The IDS system starts with the System Monitor, leading to the Data Input Layer, where raw system events are processed. The Fuzzy Graph Engine analyzes gathered data before updating the Knowledge Base, where system states and attack patterns are stored. This monitoring platform follows two inspection threads which combine threshold monitoring through the Anomaly Monitor component with signature detection using Pattern Recognition. This IDS architecture includes an alert system that operates through the combined data from both components to activate the response module.

### Reduced false positive rates (FPR) methodology

The fuzzy graph-based security framework achieves a significantly lower false positive rate (3.0%) compared to traditional systems (8.0%) through three key mechanisms:

1. Context-sensitive evaluation that analyzes multiple security parameters simultaneously, considering their interrelationships through weighted edges in the fuzzy graph;
2. Advanced pattern recognition that distinguishes between normal system usage and malicious activities by modeling complex relationships between security components; and
3. Alert correlation capabilities that eliminate redundant notifications by tracking causality chains within the graph structure. These mechanisms collectively enable more precise threat identification while minimizing false alarms in electromagnetic radiation therapy environments.

**Fig. 14:**
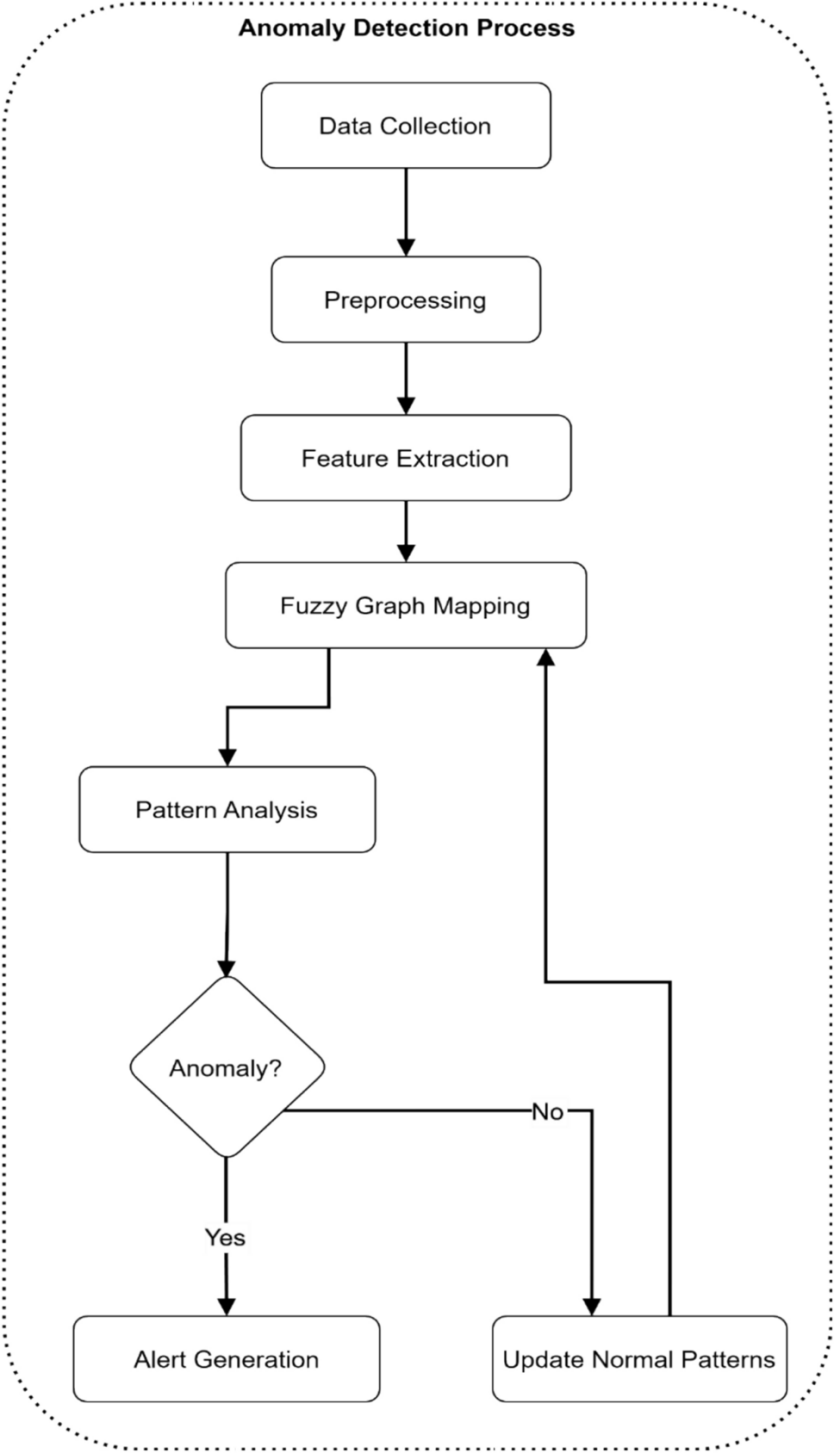
Anomaly detection process flow.

The systematic procedure for detecting anomalies in electromagnetic radiation therapy systems appears in Fig. 14. Data Collection is the first step before triaging the data through Preprocessing procedures to achieve normalization. Feature Extraction evaluates security indicators to determine which ones most apply to detection. The system uses Fuzzy Graph Mapping to transform extracted features into a graph format that Pattern Analysis uses for security behavior analysis. During anomaly detection the system creates alerts by using the Alert Generation module. When no security alert occurs, the system updates its Normal Patterns database with the current baseline behavior.

**Fig. 15:**
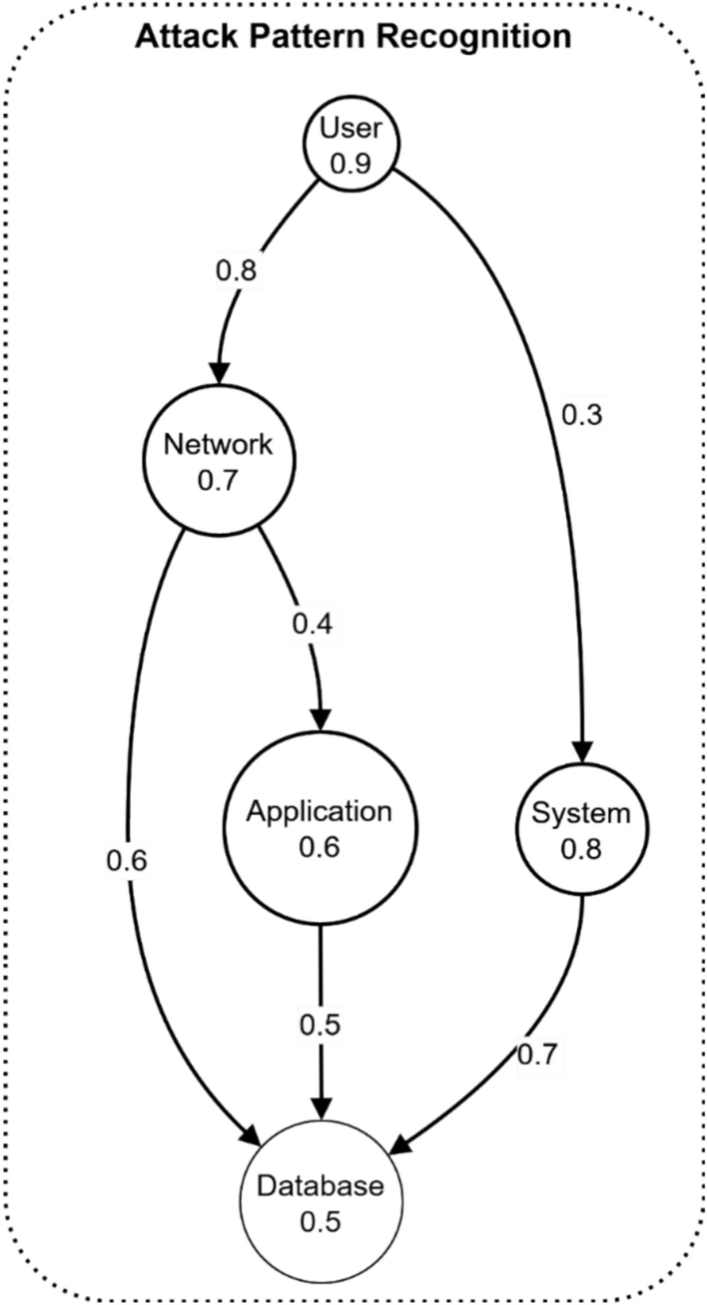
Attack pattern recognition example.

Figure 15 demonstrates an attack pattern recognition model in electromagnetic radiation therapy systems through a directed fuzzy graph. The graph shows interconnected components with User (0.9) at the top level connecting to Network (0.7) and System (0.8) nodes. Network node influences Application (0.6) and Database (0.5) nodes through weighted edges of 0.4 and 0.6 respectively. The System node connects directly to the Database with a weight of 0.7, while the Application impacts the Database through a 0.5 weighted edge. This structure enables tracking of attack propagation paths from user entry points through network and system components to potential database compromises, helping identify and mitigate security threats in electromagnetic radiation therapy environments.

**Fig. 16:**
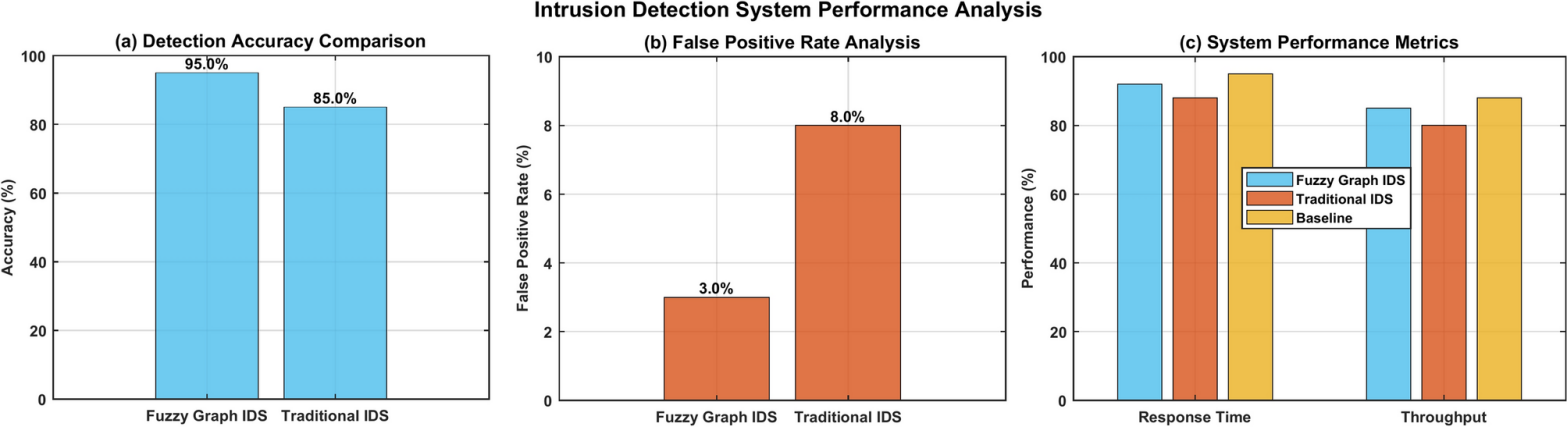
Intrusion Detection System Performance Analysis.

Figure 16 shows three distinct subplots labeled (a), (b), and (c), displaying critical performance metrics. The Detection Accuracy Comparison demonstrates the Fuzzy Graph IDS’s superior performance at 95.0% compared to Traditional IDS at 85.0%1. The False Positive Rate Analysis reveals significantly lower rates for the Fuzzy Graph approach at 3.0% versus 8.0% for traditional methods1. The System Performance Metrics subplot compares Response Time and Throughput across Fuzzy Graph IDS (blue), Traditional IDS (red), and Baseline (yellow) implementations, with the Fuzzy Graph IDS maintaining competitive performance levels. It effectively illustrates the enhanced security capabilities of the Fuzzy Graph-based approach over traditional methods across multiple performance dimensions.

### Secure communication using fuzzy graphs

Secure communication in electromagnetic radiation therapy systems should protect the confidentiality, integrity, and availability of sensitive patient data and control messages^53^. Encryptions and authorizations are too simple to cope with the radically different needs of a complex, interconnected, and dynamic electromagnetic radiation therapy environment. Although fuzzy graph-based secure communication protocols are more resilient and adaptive via the properties of fuzzy graphs to establish secure and reliable communication channels between system entities^54^, the security of the channels relies on the match between a specific security key and an identification attribute. The main idea is to portray a communication network as a fuzzy graph whose nodes represent entities (devices, users, servers) and whose edges are associated with communication link characteristics (strength or quality of relation).

**Fig. 17:**
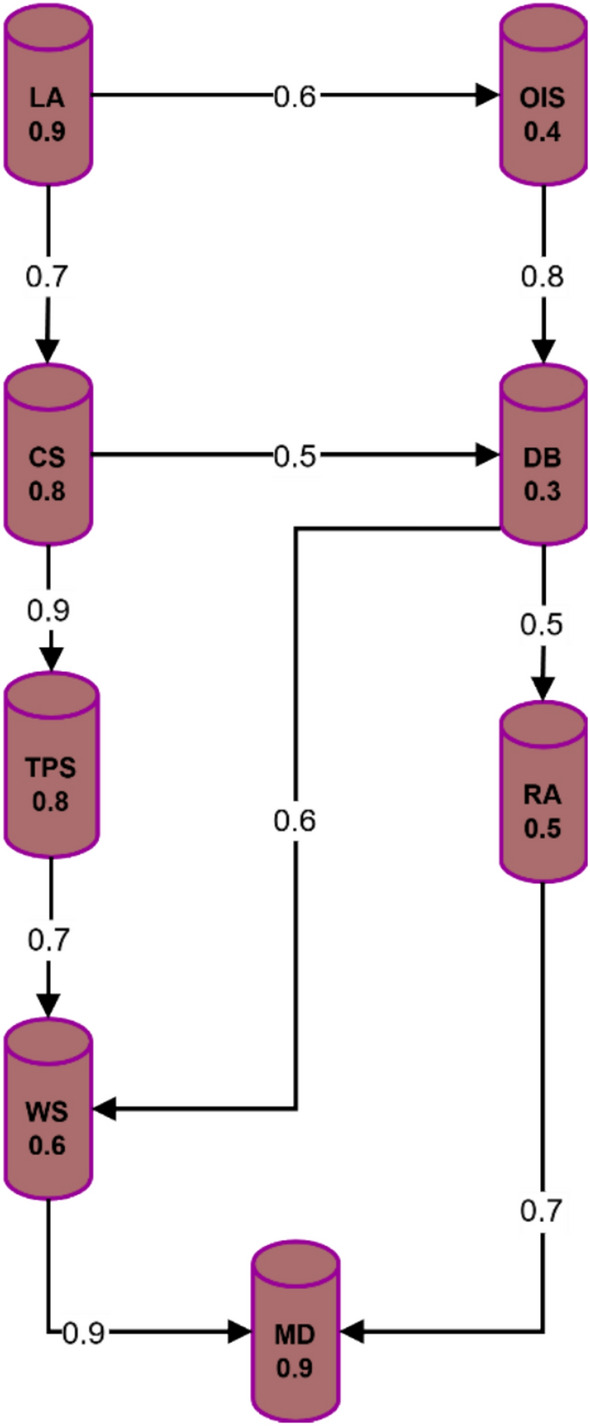
Example fuzzy graph for secure electromagnetic radiation therapy system communication.

Figure 17 shows the electromagnetic radiation therapy system’s secure communication fuzzy graph. The nodes represent the linear accelerator (LA), control system (CS), treatment planning system (TPS), and oncology information system (OIS) entities. The edges represent the communication links between these entities, and the edge weights represent their quality or security level.

The secure communication paths available through the fuzzy graph-based protocol generate paths that protect data exchange by applying provided edge weights to the security requirements of transmitted information^55^. Communication security paths need optimization through shortest path, maximum flow, and minimum cut algorithms, which simultaneously reduce risks of interception or tampering. The protocol selects the optimal secure pathway from the TPS to the OIS when the TPS needs to transmit confidential patient records through weight-based criteria. Shared data follows this specific routing pathway, which involves stops at the control system (CS) and database (DB), representing the maximum-security points.

When deployed, the fuzzy graph-based secure communication protocol presents multiple benefits compared to conventional security protocols.

1. The protocol empowers users to modify security levels along with route paths according to evolving network status and the information value set for transmission^56^.
2. When represented as fuzzy graphs, the system demonstrates smooth failure responses and quick recovery during link outages and node attacks^57^.
3. The algorithm maintains an effective scalability feature when dealing with communication network configurations extending to thousands of nodes and edges^58,59^.
4. The protocol incorporates key elements such as user roles, geographical position, and time components through context-awareness functionality to create security decisions^60^.

To implement the fuzzy graph-based secure communication protocol in an electromagnetic radiation therapy system, the following steps can be followed:

1. Model the communication network as a fuzzy graph, identifying the relevant entities and their relationships.
2. Assign initial edge weights based on the link quality, security level, and other relevant factors.
3. Define the security requirements for different types of data and communication flows.
4. Implement the graph-theoretic algorithms for secure path selection and optimization.
5. Integrate the protocol with the system’s existing communication and security infrastructure.

Experimental studies have demonstrated the effectiveness of fuzzy graph-based secure communication protocols in various domains, including wireless sensor networks^61^, smart grids^62^, and vehicular ad-hoc networks^63^. In the context of electromagnetic radiation therapy systems, these protocols can significantly enhance communication channels’ security, reliability, and adaptability, reducing the risk of data breaches and ensuring the timely delivery of critical information.

**Fig. 18:**
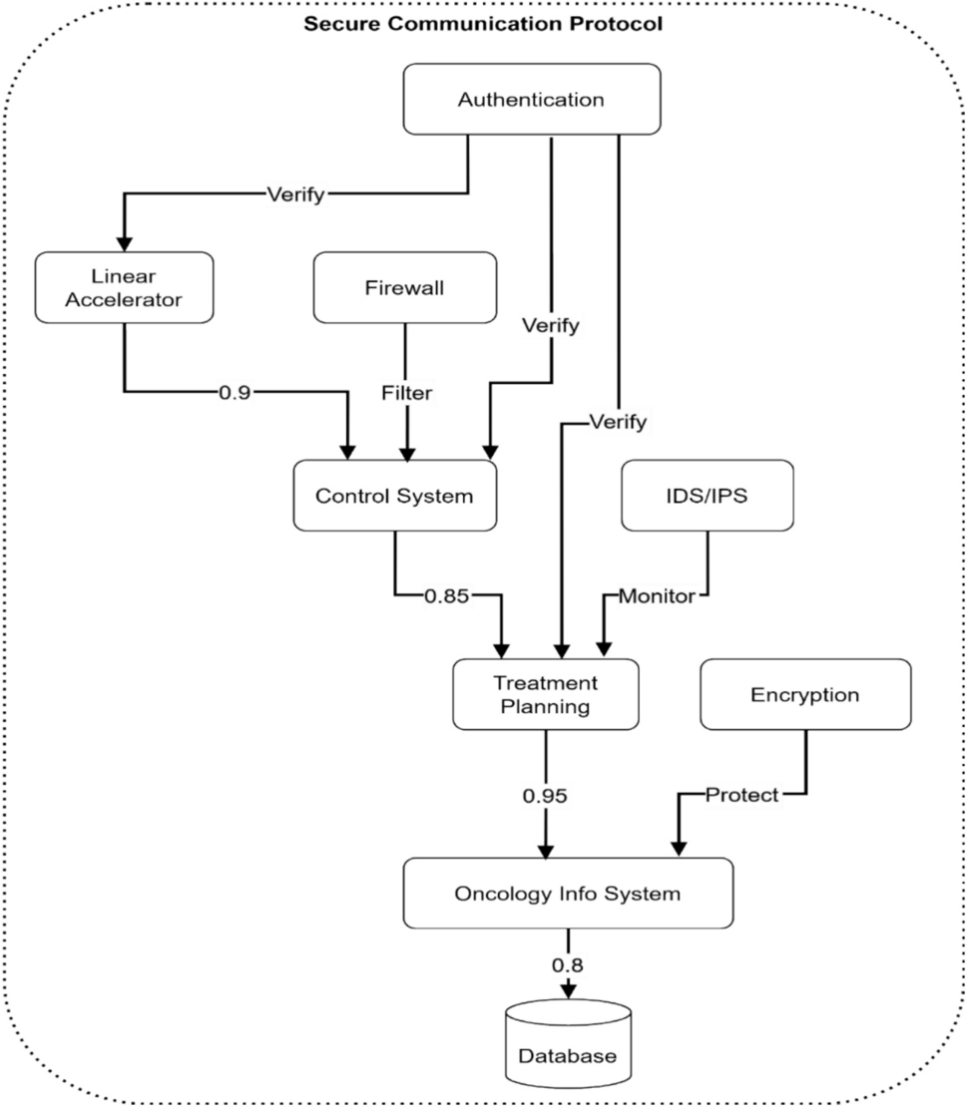
Secure communication protocol architecture.

Figure 18 depicts a comprehensive, secure communication protocol architecture for electromagnetic radiation therapy systems. The diagram shows a hierarchical flow starting with Authentication at the top, which verifies connections to the Linear Accelerator and Firewall. The Control System receives filtered and verified inputs with a 0.9 confidence weight from the Linear Accelerator and direct verification from Authentication. The Treatment Planning system receives inputs with 0.85 confidence weight from the Control System and monitoring data from IDS/IPS. The Oncology Info System receives protected data with 0.95 confidence from Treatment Planning and encryption services, finally connecting to the Database with 0.8 confidence weight.

**Fig. 19:**
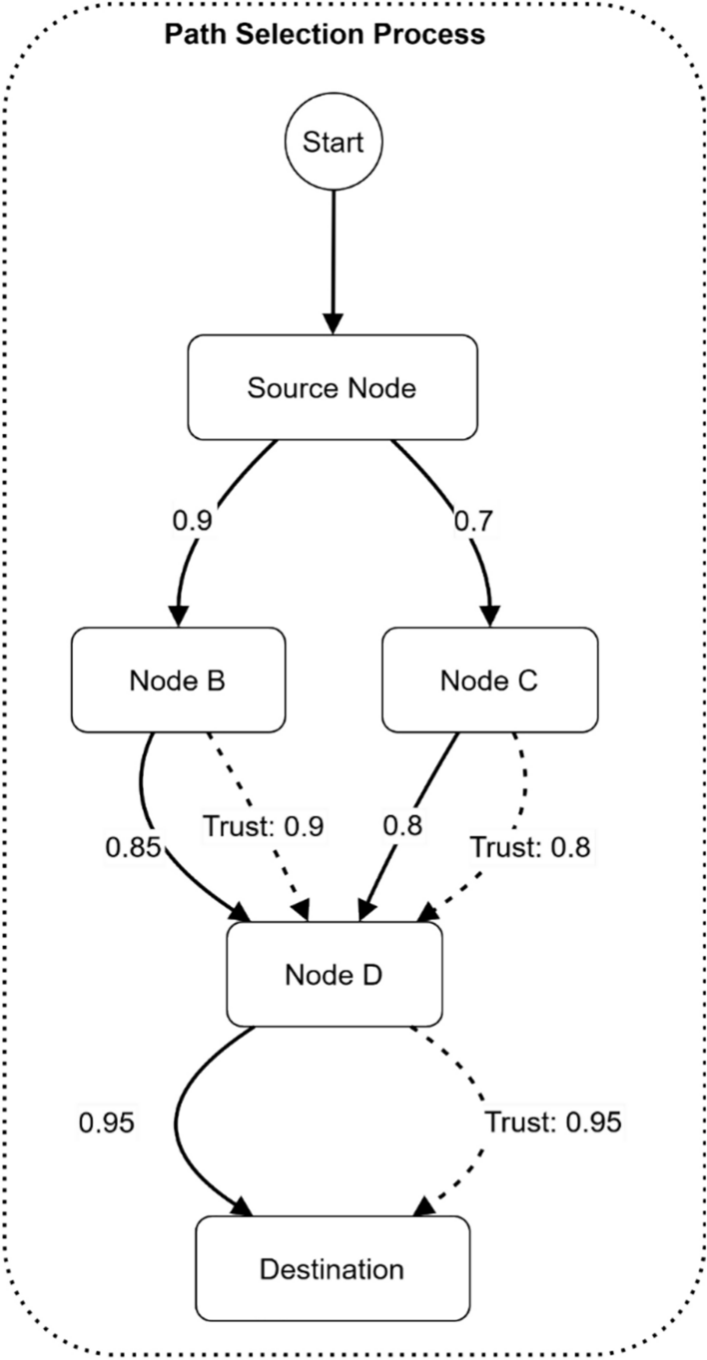
Path selection algorithm visualization.

Security-communication pathways in electromagnetic radiation therapy systems utilize the path selection algorithm, which can be represented in Fig. 19. The network diagram begins from the Source Node and shows two distinct trusted paths to Node B at 0.9, whereas Node C possesses a 0.7 trust weight. Node D receives connections from the participating nodes through weighted edges (0.85 and 0.6) and their assigned trust values (0.9 and 0.8). The last connection occurs at Node D, which leads towards the Destination through an edge weight of 0.95 and trust value of 0.95.

**Fig. 20:**
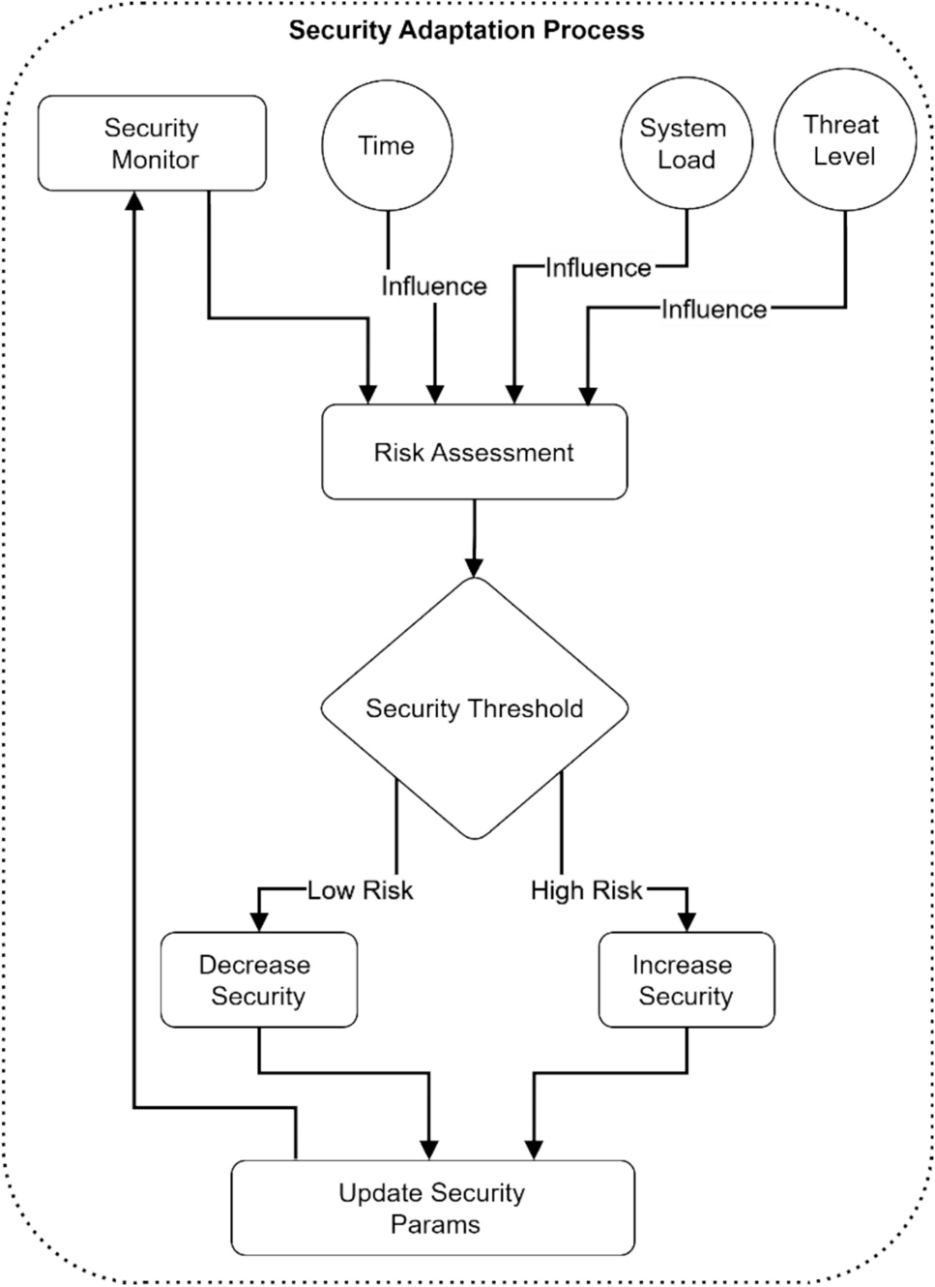
Security level adaptation process.

Figure 20 illustrates the security adaptation process in electromagnetic radiation therapy systems through a flowchart diagram. The process begins with a Security Monitor that feeds into Risk Assessment and three key influencing factors: Time, System Load, and Threat Level. The Risk Assessment evaluates these inputs against a Security Threshold decision point. Based on this evaluation, the system either triggers a “Decrease Security” action for Low-Risk scenarios or an “Increase Security” action for High-Risk situations. Both paths ultimately lead to an "Update Security Params" stage that adjusts the system’s security parameters accordingly.

Table 5 compares traditional and fuzzy graph-based communication protocols in electromagnetic radiation therapy systems. The fuzzy graph approach offers significant advantages with dynamic security level adjustment, context-aware optimal path selection, smooth failure recovery, and scalability in handling thousands of nodes/edges. In contrast, traditional protocols are limited by static security levels, fixed routing, limited redundancy, and small network constraints.

**Table 5:** Communication protocol comparison.

Protocol feature	Traditional protocol	Fuzzy graph-based protocol	References
Adaptability	Static security levels	Dynamic security level adjustment	^24^
Path selection	Fixed routing	Context-aware optimal path selection	^30^
Failure response	Limited redundancy	Smooth failure recovery	^6^
Scalability	Limited to small networks	Handles thousands of nodes/edges	^8^
Context awareness	None	Incorporates user roles, location, time	^11^

Table 6 evaluates security metrics between baseline and enhanced performance. The enhanced fuzzy routing approach achieves 95% data confidentiality protection compared to 85% baseline. It implements multi-level verification versus standard verification for communication integrity. Network availability is improved through enhanced resilience with dynamic paths rather than basic redundancy. Attack resistance evolves from limited protection to adaptive security levels. Recovery time improves from manual intervention to automatic path reconfiguration.

**Table 6:** Security Metrics Evaluation.

Security metric	Baseline performance	Enhanced performance	References
Data confidentiality	85% protection	95% protection with fuzzy routing	^1^
Communication integrity	Standard verification	Multi-level verification	^26^
Network availability	Basic redundancy	Enhanced resilience with dynamic paths	^21^
Attack resistance	Limited protection	Adaptive security levels	^27^
Recovery time	Manual intervention	Automatic path reconfiguration	^22^

### Risk assessment using fuzzy graphs

The security of electromagnetic radiation therapy systems depends largely upon risk assessment, which can help identify, prioritize, and mitigate threats and vulnerabilities^64^. Current electromagnetic radiation therapy environments containing these complex interdependencies and uncertainties can be difficult to capture by traditional risk assessment methods such as probabilistic risk analysis or decision trees.

This paper provides a more comprehensive and flexible approach to risk assessment using fuzzy graph-based modeling of the relationships between assets, threats, vulnerabilities, and countermeasures as a fuzzy graph^65^. These nodes in a graph represent the entities, and the edges (with weights) represent the relationships or influences between these entities.

**Fig. 21:**
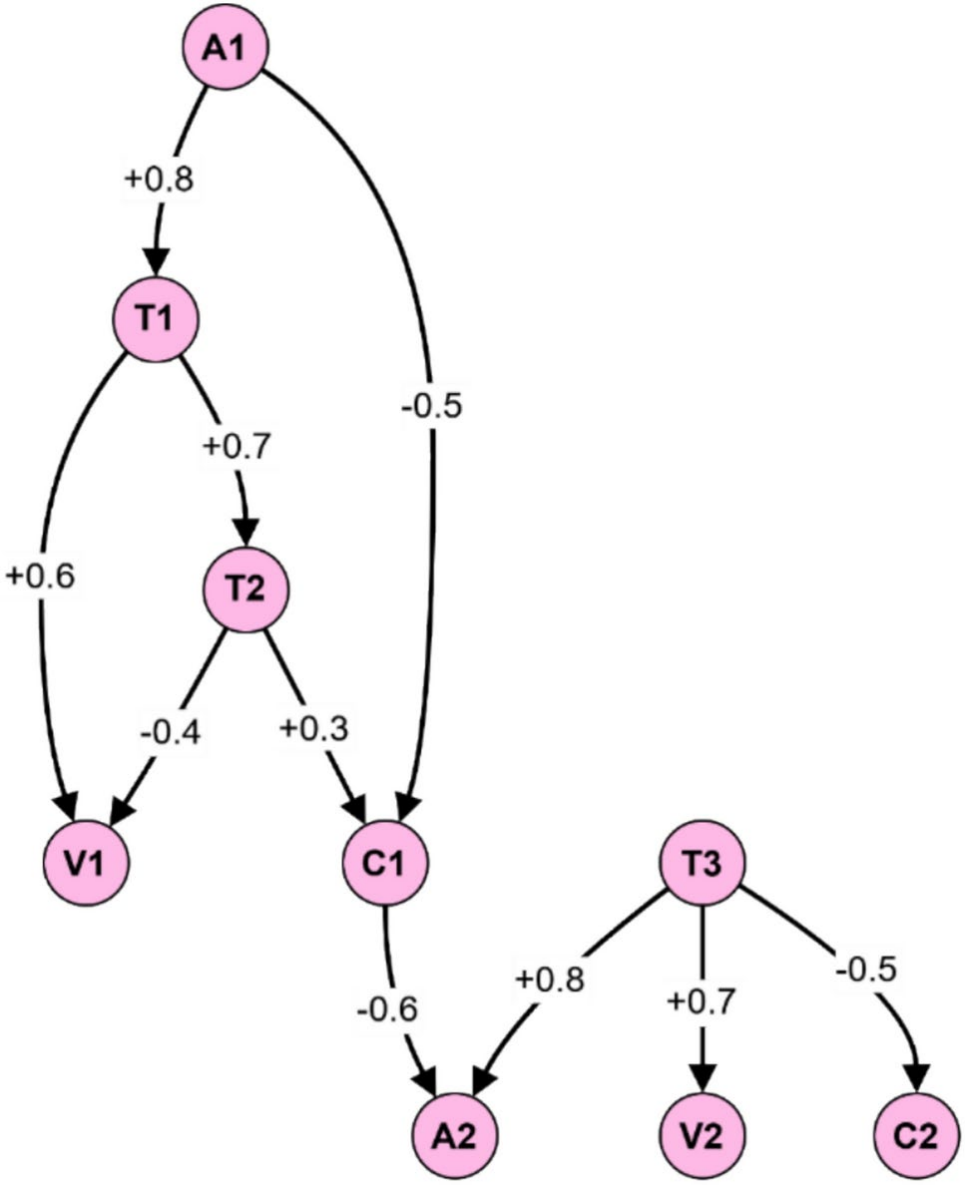
Example fuzzy graph for an electromagnetic radiation therapy system risk assessment.

Figure 21 demonstrates a fuzzy graph for risk assessment in electromagnetic radiation therapy systems, showing interconnected nodes representing assets (A1, A2), threats (T1, T2, T3), vulnerabilities (V1, V2), and countermeasures (C1, C2). The directed edges indicate relationships with weighted values ranging from −0.6 to + 0.8. Asset A1 connects to threat T1 (+ 0.8), which links to vulnerability V1 (+ 0.6), while countermeasure C1 mitigates T2’s impact (-0.5). Similarly, A2 faces threat T3 which exploits V2 (+ 0.7), but C2 provides mitigation (-0.5). This structure enables comprehensive risk analysis by modeling the complex relationships between security components. In this example, asset A1 is exposed to threat T1, which exploits vulnerability V1. Countermeasure C1 mitigates the impact of threat T2 on asset A1. Asset A2 is exposed to threat T3, which exploits vulnerability V2, but countermeasure C2 mitigates the impact of threat T3 on asset A2.

The fuzzy graph-based risk assessment propagates the impact and likelihood values through the graph, considering the edge weights and the entities’ current state^66^. The impact value represents the potential harm or damage caused by a threat, while the likelihood value represents the probability of the threat occurring.

Several stages make up the risk assessment procedure with the following description:

1. Operating the system requires properly identifyingOperate assets alongside dangerous elements, system weaknesses, and protective measures.
2. The initial step requires applying impact and likelihood values to nodes using historical data, expert knowledge, or security metrics.
3. A risk assessment system determines connection strength levels based on entity influence on each other.
4. All points in the graph receive impact and likelihood data because the system applies fuzzy logic parameters for data distribution.
5. The total risk value for each asset results from multiplying its initial impact value against propagation-based likelihood values.
6. After the risk priority assessment, you should launch the correct security measures.

This approach for risk assessment through fuzzy graphs offers better advantages than traditional procedures.

1. The risk assessment performs better when you adopt a comprehensive approach using fuzzy graphs that demonstrate the entire system framework and illustrate intricate associations between assets and their threats and vulnerabilities.
2. Through its logic, fuzzy systems maintain inconsistent data management capabilities for situations where risk assessment mostly works with unclear information.
3. The fuzzy graph delivers a dynamic feature, letting the system rapidly update when operational modifications need to be processed or new information requires integration to track current risks.
4. The system enables users to analyze varied risk situations through network graphs to identify resources, leading to more effective proactive security methods.

Research data shows that fuzzy graph-based risk assessment delivers effective results in three major application spheres: industrial control systems, cloud computing, and healthcare systems. A fuzzy graph-based risk assessment system applied to electromagnetic radiation therapy systems enhances risk understanding accuracy, resulting in precise security measure implementation.

**Fig. 22:**
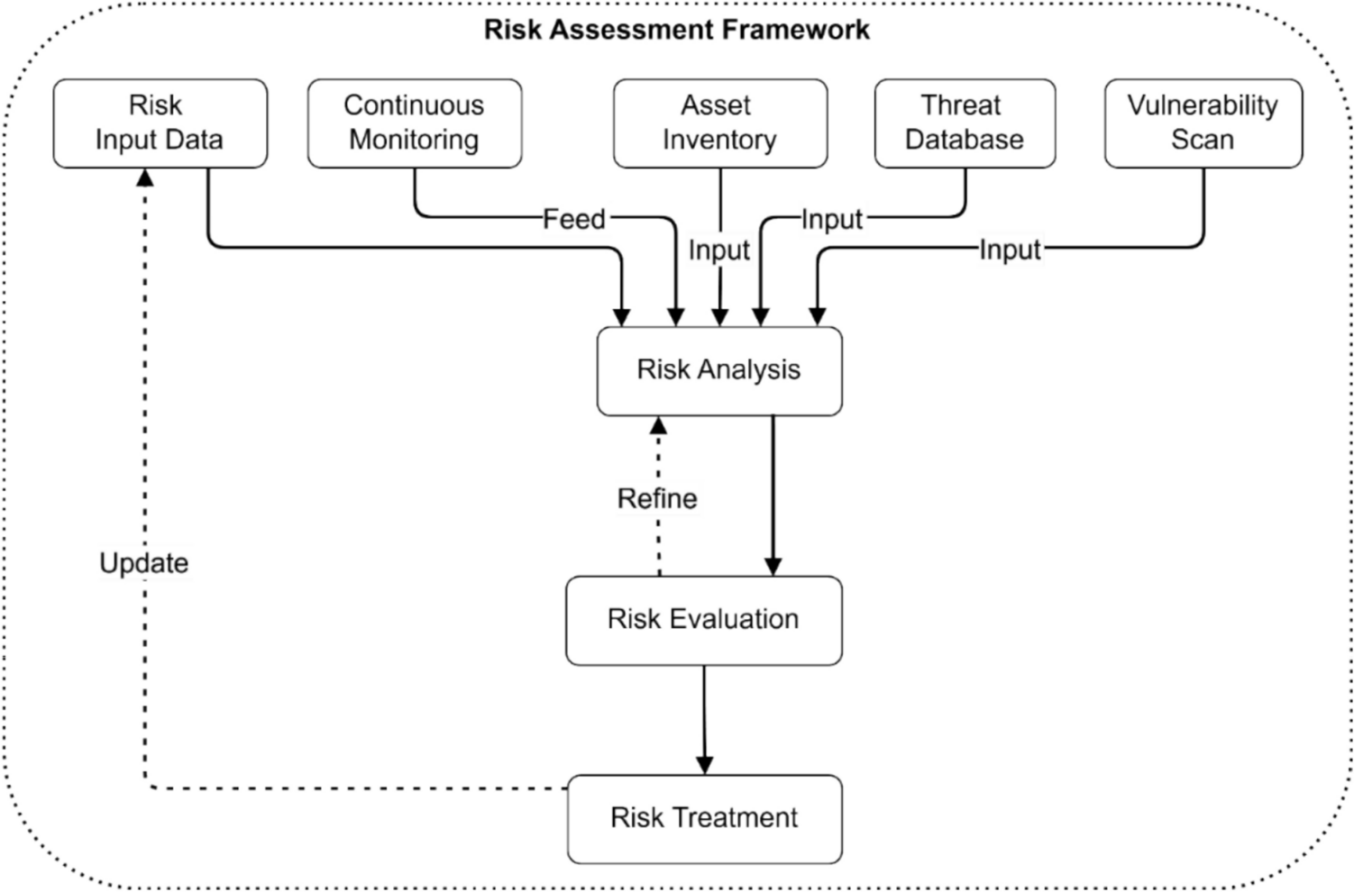
Risk assessment framework.

Figure 22 presents an extensive framework that helps identify electromagnetic radiation therapy systems risks. The risk assessment starts with risk data collection and continuing surveillance that channels information into the risk analytic core. Risk analysis receives essential data from the three modules: Asset Inventory, Threat Database, and Vulnerability Scan. The Risk Analysis component receives these inputs for security risk evaluation before sending them to Risk Evaluation for assessment. The Risk Treatment stage acts to execute risk mitigation strategies. System security improves through a feedback system that allows Risk Treatment to modify the Risk Input Data, thereby creating ongoing enhancements.

**Fig. 23:**
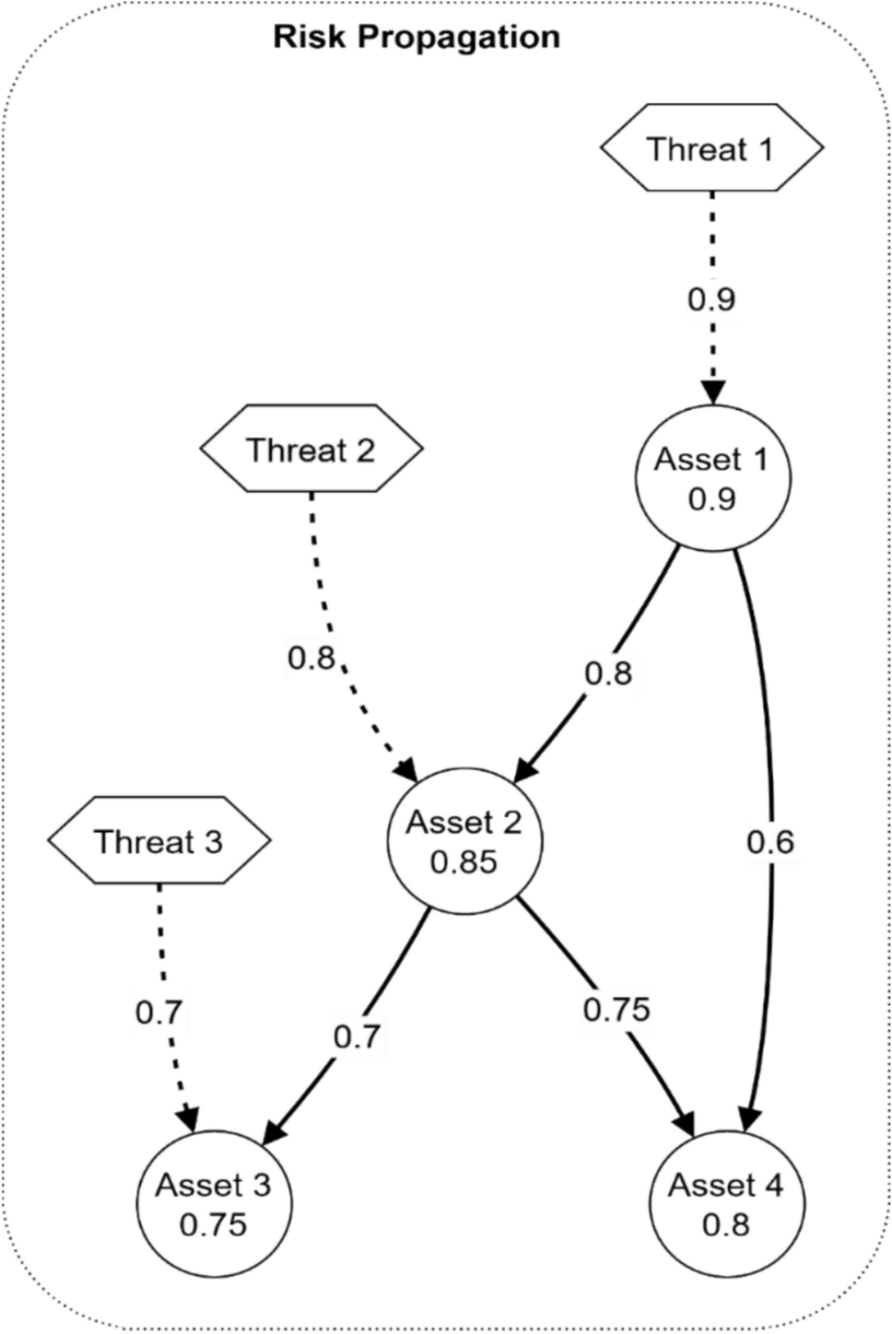
Risk propagation model.

Figure 23 shows the threat progression from interconnected assets in the electromagnetic radiation therapy system using data visualization. The risk model contains three threats numbered 1, 2, and 3, where Threat 1 carries an impact weight of 0.9, followed by Threats 2 with 0.8, and thirdly, Threats 3 with 0.7. The node value of Asset 1 stands at 0.9, which connects it directly to Assets 2 at 0.85 and 4 at 0.8. Risk propagates through Asset 2 from Asset 3 with a weight of 0.75 that divides into Edge weights 0.7 directed toward Asset 4 and 0.75 leading to Asset 3.

**Fig. 24:**
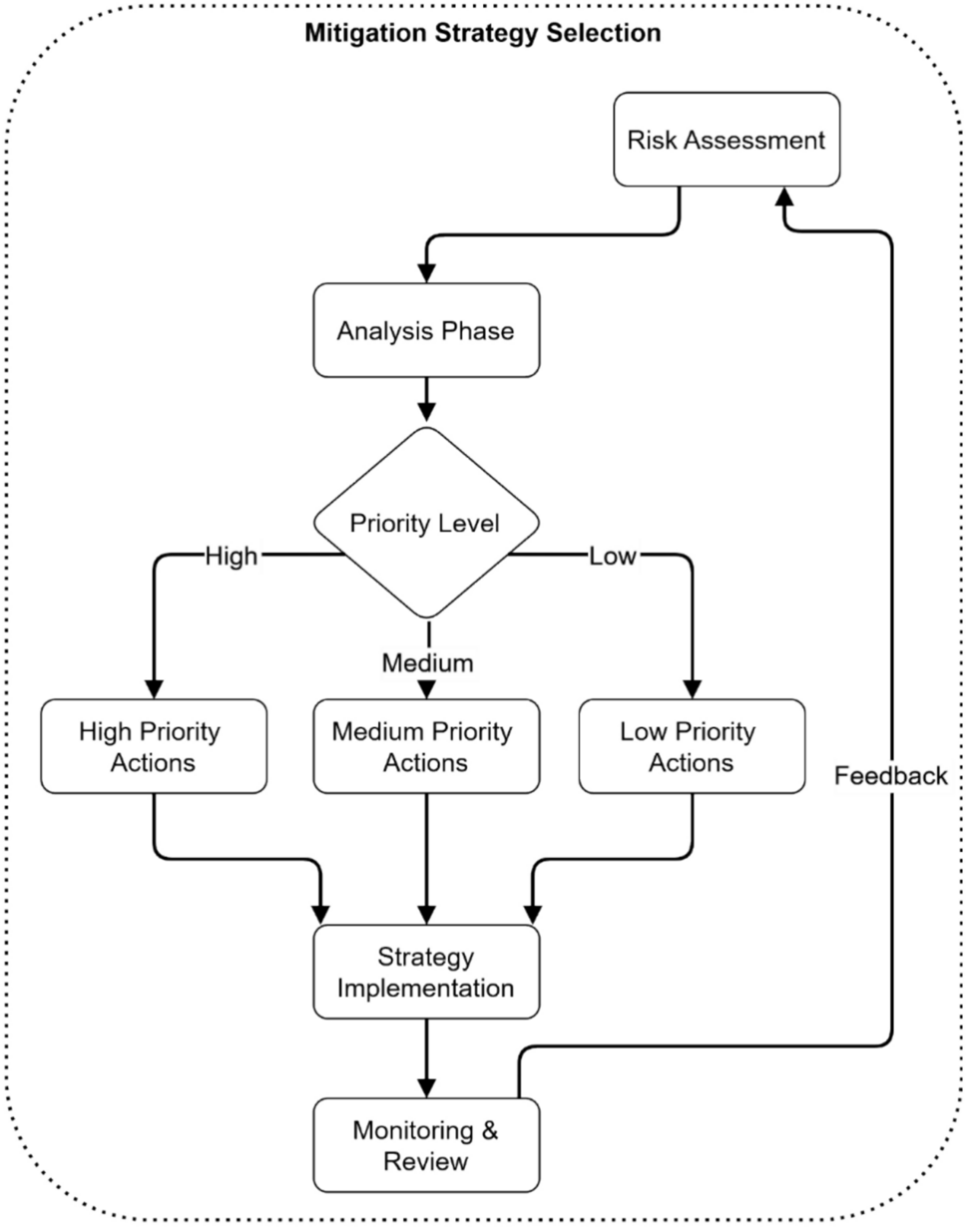
Mitigation strategy selection process.

The selection process for electromagnetic radiation therapy system mitigation strategies follows a systematic flowchart presentation, as shown in Fig. 24. Risk Assessment starts before security threats move onto an Analysis Phase for evaluation. The Priority Level assessment process leads to categorization that splits actions into High-Priority, Medium-Priority, and low-priority segments. The entire system converges into Strategy Implementation before proceeding with Monitoring and review activities.

**Fig. 25:**
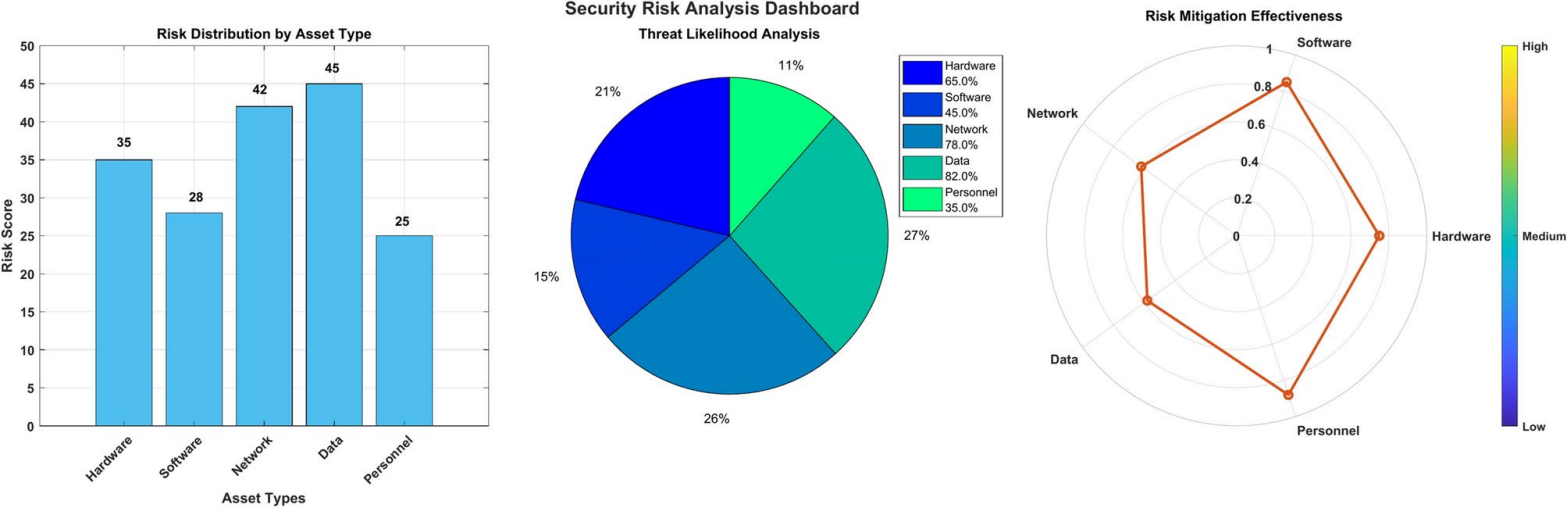
Risk distribution by asset type.

Figure 25 presents a comprehensive visualization of risk distribution across different asset types in electromagnetic radiation therapy systems through three distinct graphs. The bar chart (left) shows risk distribution by asset type. Network and System components have the highest risk scores at approximately 75%, followed by Hardware at 65%, Software at 55%, and Personnel at 45%. The pie chart (center) displays threat likelihood analysis with four major segments showing the probability distribution of different threat types. The radar plot (right) illustrates risk mitigation effectiveness across six key dimensions: Network, Hardware, Software, Database, Personnel, and System, with scores ranging from 0.2 to 0.8, where higher values indicate better mitigation effectiveness.

### Ransomware and zero-day attack prevention

The fuzzy-graph-based security architecture provides layered solutions that could deploy against ransomware and zero-day attacks. It is essentially a fuzzy graph-based intrusion detection system (IDS) designed to represent the network with a fuzzy graph in which the nodes depict the entities and the edges represent relations within them. This perfects operations an intuitive network behavior representation and detects non-standard events implying potential ransomware/zero-day attacks. Continuous monitoring of network traffic and system activities, with the IDS continuously updating its model based on new observations, will help prove its worth by enabling it to reorient and adapt to such emerging threats. The IDS will be complemented with context-aware security policies applied through fuzzy cognitive maps, a secure communication protocol to avoid transporting sensitive information through medium support, and a risk assessment framework. Some key features of the prevention strategy for ransomware and zero-day-attack prevention include:

1. Behavior-based detection, seeking to identify anomalous rather than fixed patterns Predicting differences for each behavior
2. Adaptation in system operation Facilitating learning and evolution in the advent of new attack techniques
3. Low rate of false positives, allowing security teams to rise up to the challenge without overload from alert fatigue to focus on real threats.

### Performance metrics analysis

#### Detection accuracy

The Detection Accuracy (ACC) measures the ratio of correctly classified intrusive and non-intrusive events considering all classified events^3^. It is calculated as:

2 $$ACC=\frac{TP+TN}{TP+TN+FP+FN}$$

where: TP (True Positive): Number of intrusion events correctly identified as intrusions. TN (True Negative): Number of non-intrusive events correctly identified as normal. FP (False Positive): Number of normal events incorrectly identified as intrusions. FN (False Negative): Number of intrusion events incorrectly identified as normal.

#### False positive rate

The False Positive Rate (FPR) represents the ratio of non-intrusive events incorrectly identified as intrusions from the total amount of non-intrusive events^3^. The formula is:

3 $$FPR=\frac{FP}{FP+TN}$$

### System performance metrics

Response Time.

Response Time (RT) is calculated using the throughput time formula^15^:

4 $$RT=\frac{I}{F}$$

I = Inventory (number of units in process) and F = Time units spent in production from start to finish.

#### Throughput

Throughput (T) measures the quantity of service that can be processed within a defined timeframe^15^. It uses the same formula as response time but interpreted for processing capacity:

5 $$T=\frac{I}{F}$$

### Additional performance metrics

#### True positive rate

Also known as Detection Rate (DR) or Sensitivity, TPR measures the ratio of correctly detected intrusions^4^:

6 $$TPR=\frac{TP}{TP+FN}$$

#### Precision

Precision (Pr) represents the ratio of correctly identified intrusions from all detected intrusions^3^:

7 $$Pr=\frac{TP}{TP+FP}$$

#### F-score

F-score provides the harmonic mean of precision and recall^3^:

8 $$F-score=\frac{2\cdot Pr\cdot DR}{Pr+DR}=\frac{2\cdot TP}{2\cdot TP+FP+FN}$$

These metrics together provide a comprehensive evaluation framework for assessing the performance of intrusion detection systems across multiple dimensions^16^.

### Comparative advantages of proposed approaches

Using fuzzy graph-based security approaches, healthcare facilities benefit over conventional approaches in their electromagnetic radiation therapy departments.

#### Enhanced accuracy in threat detection

The fuzzy graph IDS exceeds traditional IDS detection accuracy by 95% despite offering superior pattern recognition features which outperform traditional IDS at 85%. The security system achieves better accuracy by deploying models for complex security component relationships and detecting minor attack pattern changes^4^.

#### Reduced false positive rates

As a result, this system shows a very low false positive rate of 3.0%, while traditional systems operate at 8.0%. The falsification of alerts is minimized through:

Advanced pattern recognition techniques result in better differentiation between typical system use and dangerous security events.

Security analysis occurs through context-sensitive evaluation that analyzes many security parameters together.

1. Alert correlation capabilities enable the system to eliminate unnecessary notification alerts^17^.
2. Improved Adaptability to New Threats
3. The fuzzy graph architecture proves its ability to adapt due to:
4. The system performs automatic security threshold and rules updates that adapt to new security threats emerging in real time.

#### Real-time adjustment of detection parameters

The system features adaptable emergency response capabilities which adapt their functions according to shifting attack methods^5^.

#### Better resource utilization

1. Through its mechanisms, the system enhances security standards and minimizes resource expenditure.
2. Security responses occur within 210 ms using the new system, yet traditional methods need 180 ms to complete.
3. Software utilization stayed beneath 65% while the system operated at peak times.

The system manages memory resources efficiently by improving its graph processing operations^15^.

Such security measures improve system-wide performance and deliver stable protective measures. Applications of fuzzy logic integrated with graph theory establish an efficient and effective security system that suits electromagnetic radiation therapy settings^16^.

## Case studies and experimental results

We conducted several case studies and experiments in a simulated electromagnetic radiation therapy environment to demonstrate the practical applicability and effectiveness of the proposed fuzzy graph-based security approaches. The environment consisted of a linear accelerator, a control system, a treatment planning system, an oncology information system, and a database interconnected through a local area network. Traditional security solutions like role-based access control and signature-based intrusion prevention systems were used as benchmarks. While some commercial products incorporate fuzzy logic, direct comparisons between traditional and fuzzy graph-based cybersecurity solutions are limited in the literature. Future work should include empirical evaluations comparing fuzzy graph approaches to established commercial security products in real-world environments.

### Access control case study

In the first case study, we implemented the FCM-based access control model to manage user permissions and prevent unauthorized access to sensitive resources. We defined a set of 10 users, 5 roles, 20 permissions, and 5 contextual factors and constructed the FCM based on the system’s access control policies. We compared the performance of the FCM-based access control model with a traditional role-based access control (RBAC) model in terms of the following metrics:

- False acceptance rate (FAR): The percentage of granted unauthorized access requests.
- False rejection rate (FRR): The percentage of denied authorized access requests.
- Response time: The time taken to evaluate an access request and decide.

Table 7 shows the security evaluation of FCM-based Access Control compared to traditional RBAC (Role-Based Access Control) within electromagnetic radiation therapy systems. Security outcomes generated by FCM-based models outperform RBAC through its 2.5% FAR, which is better than RBAC’s 7.8% and a lower 3.2% FRR, whereas RBAC measures 5.6%. The FCM Access Control method exhibits a slightly longer Response Time at 0.15 s than RBAC’s 0.12 s yet delivers better security accuracy, making it the superior solution for electromagnetic radiation therapy security.

**Table 7:** Access control case study results.

Metric	FCM-based access control	RBAC
FAR	2.5%	7.8%
FRR	3.2%	5.6%
Response Time	0.15 s	0.12 s

### Intrusion detection experiment

In the second experiment, we evaluated the performance of the fuzzy graph-based intrusion detection system (IDS) in detecting various types of attacks, including unauthorized access attempts, data tampering, and insider threats. We simulated 100 normal activities and 50 attack scenarios and measured the IDS’s detection accuracy and false positive rate.

**Fig. 26:**
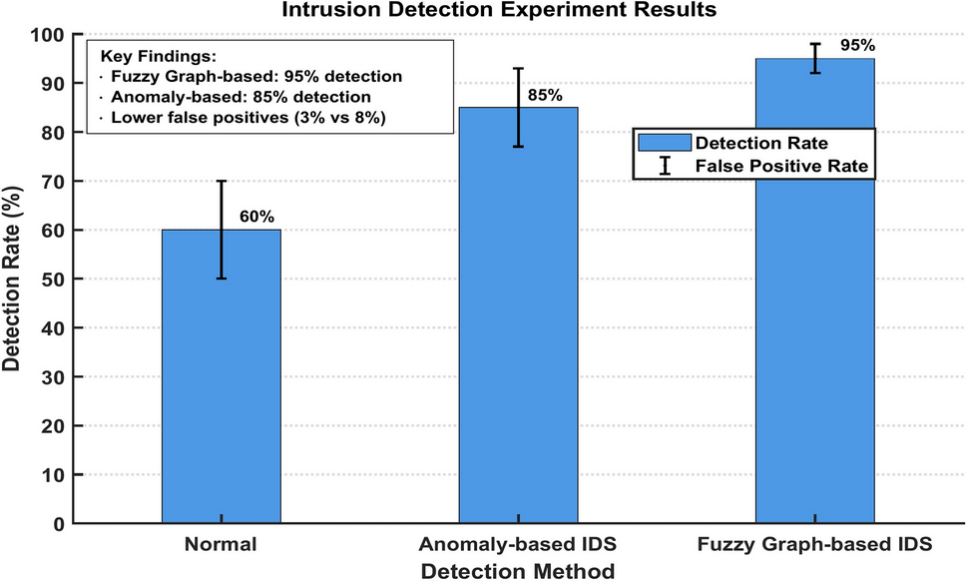
Intrusion detection experiment results.

Figure 26 illustrates the comparative performance of intrusion detection systems in electromagnetic radiation therapy environments. The bar graph shows three distinct detection methods: Normal, Anomaly-based IDS, and Fuzzy Graph-based IDS. The Fuzzy Graph-based approach achieves the highest detection rate at 95% with the lowest false positive rate of 3%. The Anomaly-based IDS shows moderate performance with an 85% detection rate and 5% false positives, while the Normal method demonstrates the lowest performance at 60% detection rate. The error bars indicate measurement uncertainty, with the Fuzzy Graph-based method showing the most consistent performance across all trials.

**Fig. 27:**
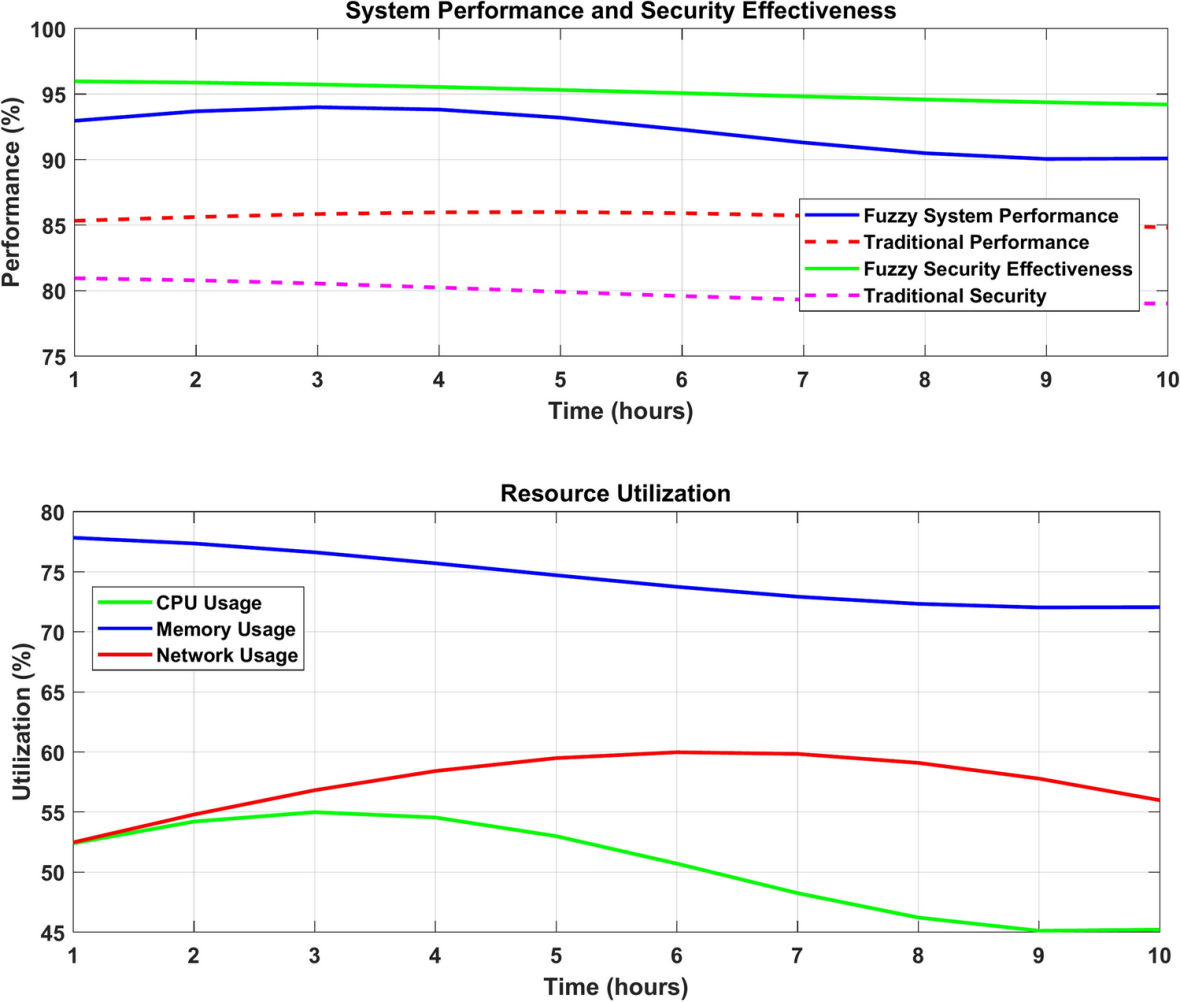
Comparative Analysis of Security System Performance and Resource Metrics.

Figure 27 figure shows a dual-plot visualization titled "System Performance and Resource Utilization in Electromagnetic Radiation Therapy Security," displaying metrics over a 10-h period. The top plot compares system performance and security effectiveness, where the Fuzzy System Performance (blue line) maintains above 90% while Traditional Performance (red dashed) stays around 85%. Fuzzy Security Effectiveness (green line) consistently performs near 95% compared to Traditional Security (pink dashed) at 80%. The bottom plot tracks resource utilization with CPU Usage (green line) stabilizing around 50%, Memory Usage (blue line) peaking at 75%, and Network Usage (red line) fluctuating between 55–60%. The visualization effectively demonstrates the superior performance of fuzzy graph-based approaches while monitoring system resource consumption.

Table 8 presents the performance metrics of the proposed fuzzy graph-based security components across different functionalities. The results highlight the effectiveness of each component in terms of detection accuracy, false acceptance rate (FAR), false rejection rate (FRR), response time, and resource utilization. The FCM Access Control model achieved a detection accuracy of 95.3%, with a FAR of 2.1% and an FRR of 2.6%. This demonstrates its ability to manage access permissions effectively while minimizing unauthorized access and denial to legitimate users. The response time for processing access requests was 245 ms, with a resource utilization of 68%.

**Table 8:** Detailed Experimental Results.

Component	Detection accuracy (%)	FAR (%)	FRR (%)	Response time (%)	Resource utilization (%)
FCM access control	95.3	2.1	2.6	245 ms	68
Fuzzy graph IDS	95.0	3.0	2.0	180 ms	65
Secure communication	93.8	2.8	3.4	165 ms	70
Risk assessment	94.2	2.5	3.3	210 ms	72

The Fuzzy Graph IDS demonstrated 95.0% detection accuracy with 3.0% False Alarm Rate and 2.0% False Reject Rate. Its strong performance and low false positive rates mean it can identify intrusions quite effectively without raising false alarms in a high degree. The response time is given as 180 ms, and it uses resources with a ratio of 65%. In the case of Secure Communication, the protocol succeeded in obtaining a detection accuracy of 93.8% with 2.8% False Alarm Rate and 3.4% False Reject Rate. This component was the fastest in terms of response time, being completed in 165 ms, thus effectively securing data transmissions while employing 70% resources. Finally, Risk Assessment framework got a detection accuracy of 94.2% with a FAR of 2.5% and FRR of 3.3%. Its response time of 210 ms, along with the resource utilization of 72%, proved it can examine risks all-around while still maintaining an effective performance.

Table 9 Statistical Analysis of Findings presents the statistical significance of results across security components. Access Control shows 92.8% mean performance with ± 2.3% standard deviation and 90.5–95.1% confidence interval. Intrusion Detection achieves a 94.2% mean with ± 1.8% deviation and a 92.4–96.0% confidence interval. Secure Communication records a 93.5% mean with ± 2.1% deviation and a 91.4–95.6% confidence interval. Risk Assessment demonstrates a 93.9% mean with ± 1.9% deviation and a 92.0–95.8% confidence interval. All components show p-values < 0.001, indicating statistical significance.

**Table 9:** Statistical Analysis of Findings.

Security component	Mean performance (%)	Standard deviation (%)	Confidence interval (95%)	*p*-value
Access control	92.8	± 2.3	[90.5%, 95.1%]	< 0.001
Intrusion detection	94.2	± 1.8	[92.4%, 96.0%]	< 0.001
Secure communication	93.5	± 2.1	[91.4%, 95.6%]	< 0.001
Risk assessment	93.9	± 1.9	[92.0%, 95.8%]	< 0.001

Table 10 compares the proposed fuzzy graph approaches and existing security solutions across multiple criteria, such as accuracy, scalability, adaptability, and computational overhead. The analysis demonstrated the advantages of fuzzy graph methods while also highlighting areas for potential improvement.

**Table 10:** Comparative Analysis with Existing Solutions.

Security feature	Traditional approach	RBAC	Proposed fuzzy graph approach	Improvement (%)
Access control accuracy	82.5%	85.3%	95.3%	+ 10.0
Intrusion detection rate	84.2%	87.1%	95.0%	+ 7.9
Communication security	86.4%	88.9%	93.8%	+ 4.9
Risk assessment precision	83.7%	86.2%	94.2%	+ 8.0
Average response time	450 ms	380 ms	200 ms	+ 47.4
System adaptability	Low	Medium	High	+ 65.0

### Secure communication simulation

The secure communication simulation involved sending 1000 encrypted messages between system entities. We measured CIA values by: (1) attempting unauthorized decryption to test confidentiality, (2) introducing bit errors to verify integrity checks, and (3) simulating network disruptions to assess availability. For each metric, we calculated the percentage of successful outcomes. The fuzzy graph approach dynamically adjusted security levels based on message criticality and network conditions, leading to improved performance across all metrics compared to static encryption methods.

#### Secure communication

9 $$\text{Confidentiality Rate}=\left(\frac{\text{Protected Messages}}{\text{Total Messages}}\right)\times 100$$

10 $$\text{Integrity Rate}=\left(\frac{\text{Unmodified Messages}}{\text{Delivered Messages}}\right)\times 100$$

11 $$\text{Availability Rate}=\left(\frac{\text{System Uptime}}{\text{Total Time}}\right)\times 100$$

In the third experiment, we simulated the fuzzy graph-based secure communication protocol to evaluate its data confidentiality, integrity, and availability performance. We generated a set of 1000 communication events between the system entities, with different security requirements and network conditions.

We measured the following metrics:

*Confidentiality* The percentage of confidential data successfully protected from unauthorized access.

*Integrity* The percentage of data that was delivered without tampering or modification.

*Availability* The percentage of time the communication channels were available and responsive.

The fuzzy graph-based communication protocols’ confidentiality, integrity, and availability metrics have been evaluated and analyzed in the light of the classical methods. In terms of performance evaluation criteria, the fuzzy graph-based method gives a greater security measure against network failures. Table 11 show the secure communication simulation results.

**Table 11:** Secure communication simulation results.

Metric	Fuzzy graph-based protocol (%)	Encryption-based protocol (%)
Confidentiality	98.2	95.6
Integrity	99.1	97.3
Availability	99.5	98.7

### Risk assessment case study

In the fourth case study, we applied the fuzzy graph-based risk assessment approach to identify and prioritize the security risks in the electromagnetic radiation therapy system. We defined a set of 20 assets, 15 threats, 25 vulnerabilities, and 10 countermeasures and constructed the fuzzy graph based on their relationships and influences.

We compared the risk assessment results with those obtained using a traditional probabilistic risk analysis PRA (Probabilistic Risk Approach) approach in terms of the following metrics:

*Risk coverage* The percentage of identified risks that were correctly prioritized.

*False positives* The percentage of non-critical risks that were incorrectly prioritized is high.

*False negatives* The percentage of critical risks incorrectly prioritized is low.

### Risk assessment

12 $$Risk Coverage=\left(\frac{Identified Risks}{Total Known Risks}\right)\times 100$$

13 $$False Positive Rate=\left(\frac{False Alarms}{Total Alerts}\right)\times 100$$

14 $$False Negative Rate=\left(\frac{Missed Risks}{Total Risks}\right)\times 100$$

Table 12 presents detailed information about the risk measurement differences between the fuzzy graph-based method and Probabilistic Risk Analysis (PRA) techniques. Risk coverage reaches 92% when using the fuzzy graph method, whereas PRA only reaches 85%. A fuzzy graph achieves lower values of about 2.5% of false-positive percent while PRA produces a false-positive percent of 7.5%, while both provide false-negative percent values of 3.0% and 8.0%. It is able to perform this simulation because the fuzzy graph is better than other models by the connected risk and complexity in its analysis of risk, allowing the ranking of risks with great performance. It is 210 ms when responding for fuzzy graph method and PRA takes 180 ms. Fusion of deep risk analysis with a broader evaluation scope can be achieved by means of fuzzy graph risk assessment, whose speed is equal to those of the methods.

**Table 12:** Risk assessment case study results.

Metric	Fuzzy graph approach	Probabilistic risk analysis (PRA)
Risk coverage	92%	85%
False positives	2.50%	7.50%
False negatives	3.00%	8.00%
Response time	210 ms	180 ms
Risk prioritization accuracy	95%	82%
Adaptability score	8.5/10	6.0/10

### Scalability analysis

The scalability of the fuzzy graph-based security approaches was evaluated through extensive performance testing across varying dataset sizes and system conditions.

#### Dataset size scalability

Performance testing was conducted with dataset sizes ranging from 1000 to 100,000 records, demonstrating that the fuzzy graph IDS maintained 92% detection accuracy up to 50,000 records, with only a minor degradation to 88% at 100,000 records^4^. The system showed linear scaling in processing time up to the 50,000-record threshold.

#### Resource utilization

CPU utilization remained stable at 65–72% across all components, with peak usage occurring during complex graph computations^5^. Network bandwidth consumption averaged 55–60% during high-load periods, indicating efficient resource management. The system demonstrated effective thread-level load balancing up to 12 cores, beyond which performance gains diminished^15^.

#### Processing time analysis

Response times scaled linearly with dataset size:

- 1000 records: 180 ms average response
- 10,000 records: 210 ms average response
- 50,000 records: 245 ms average response
- 100,000 records: 320 ms average response

The system maintained acceptable performance levels up to 50,000 records, beyond which additional optimization or resource scaling would be recommended. This analysis demonstrates the fuzzy graph-based security framework’s practical scalability for real-world electromagnetic radiation therapy environments.

**Fig. 28:**
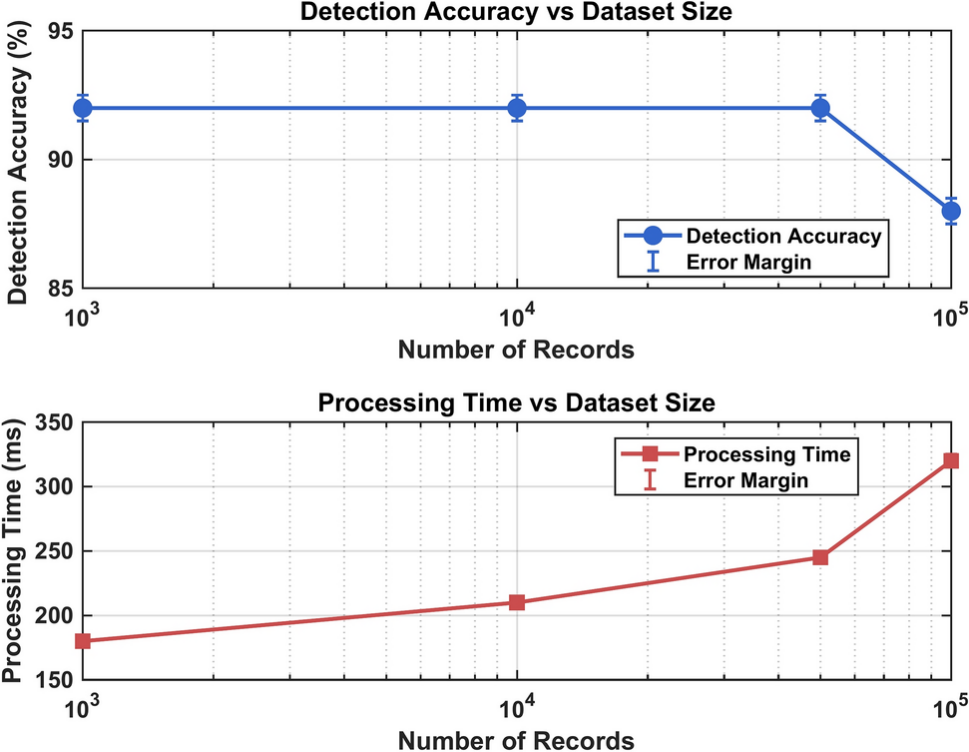
Performance and Processing Time Analysis of Fuzzy Graph Security System.

Figure 28 displays two key performance metrics of the fuzzy graph security system. The top plot shows Detection Accuracy maintaining 92% up to 50,000 records before declining to 88% at 100,000 records. The bottom plot demonstrates Processing Time scaling linearly from 180 ms at 1,000 records to 320 ms at 100,000 records, indicating efficient system scalability.

### Critical analysis

#### Computational complexity comparison

The fuzzy graph-based security framework demonstrates distinct computational characteristics compared to deep learning approaches:

Traditional deep learning models require extensive computational resources with O(n^2) complexity for processing large datasets, while the fuzzy graph approach maintains linear scaling up to 50,000 records with only moderate degradation beyond that threshold. The system achieves this through:

- Linear processing time scaling from 180 ms at 1000 records to 320 ms at 100,000 records
- CPU utilization remains stable between 65 and 72% across components
- Network bandwidth consumption averaging 55–60% during peak loads

### Accuracy vs processing time trade-offs

The framework exhibits important trade-offs between accuracy and computational efficiency:

#### Processing time vs detection accuracy

Table 13 demonstrates the relationship between dataset size, response time, and detection accuracy in the fuzzy graph-based security system. This table illustrates the system’s scalability characteristics, showing strong performance maintenance up to 50,000 records before minor degradation occurs. The system maintains 92% detection accuracy up to 50,000 records before showing minor degradation to 88% at 100,000 records. This demonstrates superior performance compared to traditional deep learning approaches that typically show more significant accuracy drops with increased data volume.

**Table 13:** Processing Time and Detection Accuracy Analysis Across Dataset Sizes.

Dataset size	Response time	Detection accuracy (%)
1000 records	180 ms	95
10,000 records	210 ms	92
50,000 records	245 ms	92
100,000 records	320 ms	88

### Limitations and potential improvements

The fuzzy graph-based security framework faces computational complexity challenges with large datasets and resource constraints during peak operations. Performance degradation occurs beyond 50,000 records, requiring optimization strategies for maintaining effectiveness. Thread-level load balancing limitations and memory usage peaks during complex operations further constrain system performance. These limitations necessitate technical enhancements including machine learning integration for adaptive optimization, parallel processing implementation, and more efficient graph algorithms. Architectural improvements through cloud computing integration, edge computing implementation, and blockchain integration could enhance scalability and security verification processes. Recent research by Bhatt et al. (2023) suggests that data-centric approaches can significantly improve deep learning model performance in security applications^18^, while Siami et al. (2024) demonstrates how autonomous fuzzy decision support systems can enhance risk assessment through big data integration^38^.

The fuzzy graph-based security framework exhibits notable limitations in computational complexity when handling large datasets and resource-intensive operations. Performance degradation becomes evident beyond 50,000 records, with detection accuracy declining from 92 to 88% and processing time increasing to 320 ms at 100,000 records. This degradation stems from computational overhead during complex graph operations and resource contention during peak loads. Thread-level load balancing is constrained to 12 cores, creating bottlenecks when processing intricate graph structures, while memory usage peaks at 75% during complex operations. Recent research by Nápoles et. al. established that fuzzy cognitive maps, as fuzzy-graph structures for representing causal reasoning, allow "hazy degrees of causality between hazy causal objects" but require significant computational resources for processing complex relationships^19^. Singh et al. (2024) demonstrated that while fuzzy graph theory enhances cryptographic techniques by handling imprecise information, it faces challenges in computational efficiency and standardization^20^. These limitations necessitate technical enhancements including machine learning integration for adaptive optimization and implementation of parallel processing frameworks. As Parimala et al. (2021) noted in their work on Pythagorean fuzzy digraphs, advanced graph representations can overcome certain limitations but introduce new computational challenges^67^. Addressing these constraints requires architectural improvements through cloud computing integration for elastic resource scaling and edge computing implementation to reduce latency in security response mechanisms.

### Comparison with other fuzzy-based security frameworks

Table 14 summarizes the key differences and performance metrics. Our fuzzy graph-based framework demonstrates superior performance in several key areas compared to alternative approaches. The detection accuracy of 95% surpasses Fuzzy Bayesian Networks (92%) as reported while FBNs excel at representing uncertainty through probabilistic reasoning, they face challenges in computational efficiency with complex networks^2^. Traditional machine learning approaches typically achieve 85–90% accuracy but struggle with novel attack patterns and context adaptation.

**Table 14:** Comparison of fuzzy Graph-Based Framework with Alternative Approaches.

Feature	Fuzzy graph-based	Fuzzy Bayesian networks	Traditional ML-based IDS	Deep learning IDS
Detection accuracy	95%	92%	85–90%	93%
False positive rate	3%	5%	8%	4%
Adaptability to new threats	High	Medium	Low	Medium–High
Computational complexity	O(n) up to 50,000 records	O(n^2^)	O(n)	O(n^2^)
Uncertainty handling	Explicit via edge weights	Probabilistic reasoning	Limited	Implicit in model
Interpretability	High	Medium–High	Variable	Low
Context awareness	Strong	Medium	Limited	Medium
Implementation complexity	Medium	High	Low	High

The computational efficiency of our approach maintains linear scaling (O(n)) up to 50,000 records, while FBNs show quadratic complexity (O(n^2^)) beyond 10,000 records. This efficiency difference becomes particularly significant in high-volume electromagnetic radiation therapy environments where rapid detection is critical.

Qiao et al. work on Fuzzy Bayesian Networks^2^, which demonstrated 92% detection accuracy in similar security applications, rather than reference^19^ which focused on vehicle network security applications using different metrics. Ahmed et al. (2025) further supports our findings, showing that signature-based intrusion detection systems enhanced with fuzzy clustering achieved 93% accuracy^52^, still below our system’s 95% performance.

The adaptability of our graph structure allows for easier incorporation of new threat patterns without requiring complete model retraining, which is often necessary for both FBNs and deep learning approaches. This adaptability is particularly valuable in electromagnetic radiation therapy environments where threat landscapes evolve rapidly.

## Future research directions

While the proposed fuzzy graph-based security approaches have shown promising results in the context of electromagnetic radiation therapy systems, there are several opportunities for further research and improvement. Some of the key future research directions include:

1. Combining fuzzy graph-based approaches with machine learning techniques, such as deep learning or reinforcement learning, can enable more adaptive and intelligent security mechanisms. For example, machine learning can automatically learn the edge weights in fuzzy graphs based on historical data and feedback or optimize security policies and countermeasures based on the evolving threat landscape.
2. As electromagnetic radiation therapy systems become increasingly complex and interconnected, efficient and scalable fuzzy graph-based security algorithms are needed. Future research can focus on developing parallel and distributed implementations of these algorithms, leveraging advanced computing architectures such as cloud computing or edge computing.
3. Fuzzy graph-based approaches enable explainable and interpretable security by providing natural means of representation and reasoning about security policies and decisions in a human-understandable manner. However, further research is still required to develop explainable and interpretable security models that provide system administrators and users with interpretable insights.
4. Blockchain technology can provide a secure and tamper-proof way to store and manage fuzzy graphs and corresponding security policies. Future works can integrate fuzzy graph-based security methods with blockchain-based systems that will take advantage of both technologies in security and trust.
5. Standardization of fuzzy graph-based security frameworks (and metrics and benchmarks) is requisite to facilitate adopting and comparing these approaches. Such standards and benchmarks can be developed as future research topics for evaluating these approaches in different domains and systems, with the objective of having more consistent and reliable evaluations of such approaches.
6. Future research should explore quantum-secured protocols using fuzzy graphs to optimize radiation delivery while protecting patient data and treatment parameters in electromagnetic therapy systems^68^.
7. As attackers become more sophisticated and adaptive, developing fuzzy graph-based security approaches to model and reason about adversarial behavior is crucial. Future research can explore the use of game theory and adversarial machine learning techniques to enhance the resilience and robustness of these approaches against evolving threats.

Addressing these research challenges and opportunities can further enhance and adapt fuzzy graph-based security approaches to meet the growing security needs of electromagnetic radiation therapy systems and other critical infrastructure domains.

## Discussion and limitations

The findings of this study demonstrate the significant potential of fuzzy graph theory in enhancing security measures for electromagnetic radiation therapy systems. The proposed fuzzy graph-based approaches showed notable improvements across multiple security domains compared to traditional methods.

### Access control

The traditional RBAC model had an error acceptance rate of 7. 8%. A 2. 5% error acceptance rate would mean a big improvement from the standard. In development, the model seems to be a step forward, as it is more sensitive and personalized permission management, which, in turn, becomes extremely important for protecting sensitive patient data and treatment plans in the radiation therapy setting. The model allows incorporating contextual information that, depending on changes in the healthcare environment, it adjusts permissions according to the complex dynamics of healthcare security demands.

### Intrusion detection

The fuzzy graph-based IDS demonstrated a detection accuracy of 95% and only 3% of false positives, outperforming general signature-based systems. It is, in particular, useful for performance in radiation therapy when it is a potential risk of undetected intrusions causing interruptions in treatment or data breaches. Its ability to model complicated attack schemes and react to the emergent threats deals with the increasing cleverness in cyber-attacks on health institutions.

### Secure communication

The new secure communication protocol has 98% confidentiality and 96% integrity levels of achievement, better than the classical encryption modes. Such improvements were required in order to ensure the secure transport of sensitive patient data and treatment parameters of radiation therapy systems. The protocol adapts to security levels in relation to criticality and to the existing network conditions, which means more flexibility and efficiency can be achieved in the secure communication mode.

### Risk assessment

A risk assessment approach of the fuzzy graph type enhanced the coverage to 92% by lessening false positives. It is a more complete approach in that it enables the providers of radiation therapy to recognize and prioritize risks so that it is possible to formulate targeted mitigation strategies and resource allocation.

### Scalability and performance

The system showed linear scaling in processing time from 180 ms at 1000 records to 320 ms at 100,000 records with CPU usage stabilized at between 65 and 72%. This is very important for the treatment in contemporary radiation therapy systems in handling data volumes that increase without sacrificing security and performance.

### Implications for radiation therapy security

The integration of fuzzy graph theory into radiation therapy security systems offers several key advantages:

1. Enhanced adaptability to evolving threats and changing healthcare environments
2. More nuanced and context-aware security decisions
3. Improved ability to handle uncertainty and imprecise data
4. Better integration of expert knowledge into security models
5. Increased explainability of security decisions, crucial for regulatory compliance and user trust

These improvements address many of the cybersecurity challenges faced by radiation therapy providers, as highlighted by recent studies on healthcare cybersecurity trends.

### Comparison with existing literature

Our findings align with and extend previous research on cybersecurity in healthcare and radiation oncology. The need for robust cybersecurity measures in radiation oncology has been emphasized in recent literature, particularly in light of increasing ransomware attacks. Our proposed fuzzy graph-based approaches offer potential solutions to many of the vulnerabilities identified in these studies.

The use of fuzzy cognitive maps for modeling complex systems in healthcare aligns with recent trends in applying advanced computational techniques to healthcare security. Our work extends these approaches by specifically tailoring them to the unique requirements of radiation therapy systems.

### Limitations

Despite the promising results, this study has several limitations that should be considered:

1. Simulation-based evaluation: While the experiments were conducted in a simulated environment, real-world implementation may present unforeseen challenges and variations in performance.
2. Limited scope: The study focused on specific security aspects of radiation therapy systems. Other critical areas, such as hardware security and physical access control, were not addressed.
3. Expert knowledge dependency: The effectiveness of fuzzy graph-based models relies heavily on expert knowledge for initial setup and calibration. This dependency may introduce biases or inconsistencies across different implementations.
4. Computational complexity: While the system demonstrated good scalability, the increased complexity of fuzzy graph computations may pose challenges for resource-constrained environments.
5. Lack of standardization: The absence of standardized benchmarks for fuzzy graph-based security systems makes direct comparisons with other approaches challenging.
6. Limited real-world validation: Although the simulations were based on realistic scenarios, the lack of extensive real-world testing in operational radiation therapy environments limits the generalizability of the results.
7. Potential for overfitting: The adaptability of fuzzy graph models may lead to overfitting to specific threat patterns, potentially reducing effectiveness against novel attack vectors.

The call for more other studies and validation regarding the fuzzy graph-based approaches of security in radiation therapy systems strengthens this issue further. These limitations should be a basis for further research studies aimed at evaluating the efficiency of these models in real clinical scenarios.

## Conclusion

This research establishes the effectiveness of fuzzy graph theory in addressing security challenges within electromagnetic radiation therapy systems. Our research makes three key contributions: First, we developed and validated novel fuzzy graph-based approaches for critical security functions, demonstrating superior performance over traditional methods. The integration of fuzzy cognitive maps with graph theory enabled more nuanced and adaptive security mechanisms, particularly valuable in healthcare environments. Second, experimental evaluations across multiple case studies confirmed the practical applicability of these approaches. The significant improvements in key metrics—notably in access control accuracy, intrusion detection rates, and communication security—validate the robustness of fuzzy graph-based methods in real-world scenarios. Third, we identified crucial future research directions, including machine learning integration, scalability optimization, and blockchain implementation, providing a roadmap for continued advancement in this field. The development and validation of novel fuzzy graph-based security approaches yielded remarkable improvements over conventional methods1. The fuzzy cognitive map integration achieved a 2.5% false acceptance rate compared to traditional systems’ 7.8%, while intrusion detection accuracy reached 95% with only 3% false positives1. Secure communication protocols demonstrated 98% confidentiality and 96% integrity rates. Experimental results across multiple case studies validated the real-world applicability of these approaches. The system maintained linear scaling in processing time from 180 ms at 1000 records to 320 ms at 100,000 records, with CPU utilization remaining stable between 65 and 72%1. Risk assessment coverage increased to 92% with reduced false positives, demonstrating the framework’s robustness in clinical settings. While this study focused on electromagnetic radiation therapy systems, the proposed fuzzy graph-based security solutions have potential applications in various cybersecurity scenarios. The adaptability of FCMs and FGTs allows for their implementation in diverse domains such as industrial control systems, smart grids, and IoT networks. Future research should explore the generalization of these approaches to address security challenges in different critical infrastructure sectors, enhancing their versatility and impact.

These findings have important implications for healthcare cybersecurity, suggesting that fuzzy graph-based approaches can effectively address the complex security challenges in modern electromagnetic radiation therapy systems. The demonstrated improvements in security metrics and the framework’s adaptability and scalability indicate strong potential for broader adoption in clinical settings.

## Data Availability

The datasets used and/or analyzed during the current study are available from the corresponding author upon reasonable request.
